# The R-RAS2 GTPase is a signaling hub in triple-negative breast cancer cell metabolism and metastatic behavior

**DOI:** 10.1186/s13045-025-01693-3

**Published:** 2025-04-12

**Authors:** Claudia Cifuentes, Lydia Horndler, Pilar Grosso, Clara L Oeste, Alejandro M. Hortal, Jennifer Castillo, Isabel Fernández-Pisonero, Alberto Paradela, Xosé Bustelo, Balbino Alarcón

**Affiliations:** 1 Immune System Development and Function Program, Centro Biología Molecular Severo Ochoa, Consejo Superior de Investigaciones Científicas, Universidad Autónoma de Madrid, Madrid, 28049 Spain; 2 Savana, S.L., Calle Gran Vía 30, Madrid, 28013 Spain; 3 University Hospital Miguel Servet, P.º de Isabel la Católica, 1-3, Zaragoza, 50009 Spain; 4 Proteomics Unit, Consejo Superior de Investigaciones Científicas, Centro Nacional de Biotecnología, Universidad Autónoma de Madrid, Madrid, 28049 Spain; 5 Centro de Investigación del Cáncer, Instituto de Biología Molecular y Celular del Cáncer, and Centro de Investigación Biomédica en Red de Cáncer (CIBERONC), CSIC-Universidad de Salamanca, Campus Unamuno s/n, Salamanca, 37007 Spain

**Keywords:** Triple negative breast cancer, Postpartum-associated breast cancer, CD44, Interaction with the extracellular matrix, Cell migration and invasion, Metastasis, Solute carriers, Large neutral amino acids transporter, PI3K pathway, mTOR pathway

## Abstract

**Background:**

Recent research from our group has shown that the overexpression of the wild-type RAS-family GTPase *RRAS2* drives the onset of triple-negative breast cancer (TNBC) in mice following one or more pregnancies. This phenomenon mirrors human TNBC, where *RRAS2* is overexpressed in approximately 75% of cases, particularly in tumors associated with the postpartum period. These findings underscore the relevance of R-RAS2 in TNBC development and progression.

**Methods:**

We conducted RNA sequencing on tumors derived from conditional knock-in mice overexpressing human wild-type *RRAS2* to identify the somatic mutation landscape associated with TNBC development in these mice. Additionally, we developed a TNBC cell line from *RRAS2*-overexpressing mice, enabling loss-of-function studies to investigate the role of R-RAS2 in various pathobiological parameters of TNBC cells, including cell migration, invasiveness, metabolic activity, and metastatic spread. Furthermore, proteomic analysis of a freshly isolated tumor identified plasma membrane receptors interacting with R-RAS2.

**Results:**

Our findings demonstrate that TNBC driven by *RRAS2* overexpression exhibits a pattern of somatic mutations similar to those observed in human breast cancer, particularly in genes involved in stemness, extracellular matrix interactions, and actin cytoskeleton regulation. Proteomic analysis revealed that wild-type R-RAS2 interacts with 245 membrane-associated proteins, including key solute carriers involved in cell metabolism (CD98/LAT1, GLUT1, and basigin), adhesion and matrix interaction proteins (CD44, EpCAM, MCAM, ICAM1, integrin-α6, and integrin-β1), and stem cell markers (β1-catenin, α1-catenin, PTK7, and CD44). We show that R-RAS2 regulates CD98/LAT1 transporter-mediated mTOR pathway activation and mediates CD44-dependent cancer cell migration and invasion, thus providing a mechanism by which R-RAS2 promotes breast cancer cell metastasis.

**Conclusions:**

R-RAS2 associates with CD44, CD98/LAT1, and other plasma membrane receptors to regulate metabolic activity, actin cytoskeleton reorganization, cell migration, invasion, and distant metastasis formation in TNBC. These findings establish R-RAS2 as a central driver of TNBC malignancy and highlight its potential as a promising therapeutic target, particularly in aggressive, postpartum-associated breast cancers.

**Supplementary Information:**

The online version contains supplementary material available at 10.1186/s13045-025-01693-3.

## Background

Breast cancer (BC) is the most frequently diagnosed cancer in women worldwide and it is the leading cause of cancer-related deaths, the majority of which result from metastatic disease (https://www.who.int/news-room/fact-sheets/detail/breast-cancer). BC is a heterogeneous disease that is classified histologically into two main types: ductal and lobular. Ductal carcinomas originate in the mammary ducts, while lobular carcinomas arise from the milk-producing lobules [1]. Both types differ in epidemiology, molecular alterations, clinicopathologic aspects and natural history. Invasive ductal adenocarcinomas constitute the majority (≈ 80%) of newly diagnosed BCs. In molecular terms, BC is classified according to the expression of the estrogen receptor (ER), progesterone receptor (PR) and epidermal growth factor receptor 2 (HER2, also known as ERBB2), and by the proportion of mitotic (Ki67^+^) cells [2]. Accordingly, the main molecular types of BC are luminal A, luminal B, HER2^+^ and triple-negative (TNBC) breast cancer. This classification has important prognostic and therapeutic implications, as it reflects the sensitivity of these tumors to hormone and/or immunotherapy. TNBC is generally considered the type with worse outcomes due to its histological status, rate of proliferation, capacity to metastasize, and its insensitivity to hormone and antibody therapies [1, 2]. TNBC accounts for 10–20% of all BCs and patients diagnosed with TNBC have a 5-year survival rate 8-16% lower than that of patients with hormone receptor-positive BC [3]. However, under the TNBC classification, based primarily on immunohistochemical markers there is a heterogenous assortment of gene expression profiles that influence the response to standard chemotherapeutic treatments and to immunotherapy [4]. One of the most used classifications is this of Lehmann et al. who divided TNBC into six different subtypes according to gene expression and response to standard treatments: the immunoregulation (IM), the mesenchymal stem-like (MSL), the mesenchymal (M), the basal-like 1 (BL1), the basal-like 2 (BL2), and the luminal androgen receptor (LAR) [5]. The LAR subtype is more sensitive to anti-AR therapies. The BL1 subtype shows increased activity in growth factor signaling, cell cycle progression and DNA damage regulation. The BL2 subtype is also characterized by abundant growth factor signaling and presence of myoepithelial cell markers. BL1 and BL2 show increased susceptibility to cisplatin compared to others whilst the BL1 subtype shows better responses to PARP inhibitors and BL2 to mTOR and growth factor inhibitors [4–6]. The M subtype has highly activated cell migration-related signaling pathways (regulated by actin), extracellular matrix–receptor interaction pathways, and differentiation pathways (Wnt pathway, anaplastic lymphoma kinase pathway, transforming growth factor (TGF)-β signaling) and is therefore also called metaplastic breast cancer. The IM subtype has significantly enriched immune cell-associated genes and signal transduction pathways, and therefore it is recommended to use PD1, PDL1, CTLA-4, and other immune checkpoint inhibitors for the treatment of patients with this type of TNBC [6]. The M subtype has sarcoma-like or squamous epithelial cell-like tissue characteristics and is prone to develop resistance to chemotherapeutic drugs. Therefore, M-subtype patients might be treated with mTOR inhibitors or drugs targeting epithelial–mesenchymal transition [6]. The MSL subtype expresses high levels of stemness-related genes, HOX genes, and mesenchymal stem cell-specific markers. It is speculated that the MSL subtype patient may be treated with PI3K inhibitors, Src antagonists, or antiangiogenic drugs. Another molecular classification of TNBC into 4 subtypes was proposed by Burstein et al. [7]. Interestingly, in this classification, MSL and M subtypes were grouped into a single mesenchymal (MES) subtype and this type was associated with a poorer distant metastasis-free survival at 5 years compared to other subtypes, likely associated with increased expression of cellular motility genes leading to increased metastasis [5].

Although childbearing is known to have a long-term protective effect against breast tumor development, studies of BC incidence in young women demonstrate a transient risk in tumor development in the years immediately following childbirth [8]. Compared with nulliparous women, parous women had a hazard ratio (HR) for breast cancer that peaked about 5 years after birth (HR, 1.80 [95% CI, 1.63 to 1.99]) before decreasing to 0.77 (CI, 0.67 to 0.88) after 34 years [8]. The association crosses over from positive to negative at about 24 years after giving birth. Post-partum BC is associated with worse survival and a higher risk of metastasis than BC diagnosed in age-matched control women. This difference could be related to a higher incidence of TNBC in post-partum BC individuals [9, 10]. The transient increase in the risk of BC associated with recent pregnancy could be related to the significantly enhanced proliferation of mammary epithelial cells or post-partum/post-lactational mammary tissue involution induced by pregnancy-related hormones. Involution may mimic aspects of wound healing, including the presence of activated fibroblasts, extracellular matrix (ECM) deposition and elevated matrix metalloproteinase (MMP) levels, thus resembling a pro-tumorigenic wound environment [11].

A number of signaling pathways are particularly relevant in BC cells: the ER-signaling and HER2-signaling pathways for ER^+^ and HER2^+^ tumors, as well as the Wnt/β-catenin and PI3K/Akt/mTOR pathways. Indeed, alterations to genes that participate in these pathways have been detected in BC [12]. Besides HER2 (ERBB2), other ErbB receptor family members, including ERBB4/HER4, play key roles in BC. While TNBC lacks HER2 overexpression, high ERBB4 levels have been linked to poor prognosis [13]. In addition to ERBB members other important family of membrane receptor tyrosine kinases relevant in BC is the Ephrin receptor family, of which EphA2 has emerged as a major player in BC tumorigenesis, including TNBC [14].

Other membrane receptors that do not belong to the receptor tyrosine kinase superfamily have been found to play fundamental roles in BC transformation and metastasis. One of those is the type II membrane protein CD98 (CD98hc, 4F2), encoded by the *SLC3A2* gene, that forms covalent associations with other proteins including L-type amino acid transporters (LATs) for which CD98 serves as a chaperone. One of the most important partners of CD98hc is LAT1 (CD98lc), encoded by the *SLC7A5* gene, which plays a fundamental role allowing the transport of essential large amino acids like leucine and isoleucine. This transport serves in addition an important role in the regulation of cell metabolism via the activation of the mTORC1 pathway. *SLC7A5* (LAT1) is generally overexpressed in cancer cells [15, 16], including breast cancer of luminal and TNBC types, in which LAT1/CD98 overexpression serves as a prognostic marker [17, 18]. Indeed, LAT1 targeting with specific inhibitors is being proposed as a therapeutic strategy for TNBC and also for estrogen deprivation-resistant luminal BC [19]. In addition to forming covalent heterodimers with LATs, CD98hc interacts with other membrane receptors that play important roles in cancer cell adhesion, metabolism and migration, such as CD147 (basigin, emmprin) and β1 integrins [15, 16].

Another non-kinase, single-pass membrane receptor which has elicited increased interest in BC is CD44. CD44 is expressed by embryonic stem cells and a variety of cell types, with multiple isoforms depending on the tissue, and is also heightened in certain cancer cell subpopulations, serving as a molecular marker for cancer stem cells (CSC) [20–22]. The primary ligand for CD44 is hyaluronic acid (HA), a plentiful component of the extracellular matrix (ECM). HA binds to CD44, causing conformational changes that enable the binding of adaptor proteins or cytoskeletal elements to intracellular domains, subsequently activating multiple signaling pathways that drive cell proliferation, adhesion, migration, and invasion [23]. Cancer cells that undergo epithelial to mesenchymal transition (EMT) acquire stem cell-like characteristics and exhibit increased CD44 expression accompanied by enhanced invasiveness [24].

The process of EMT involves the activation of the Wnt signaling pathway which among other effects downregulates E-cadherin expression, which is fundamental to maintain epithelial cell juntions, and upregulate the expression of the mesenchymal cell marker vimentin [25, 26]. In TNBC, the mesenchymal-like (M and MSL) subtypes revealed a profile of EMT related pathways, which included the Wnt/β-catenin-dependent signaling pathway. Furthermore, mutations of Wnt/β-catenin-dependent signaling have been found in metaplastic BC (corresponding to the mesenchymal subtype) [27]. In addition to EMT, Wnt signaling plays a pivotal role in maintaining BC stem cells and their self-renewal ability. Indeed, CD44 and protein C receptor (ProCr), considered as markers of stem-like cells are both targets of Wnt β-catenin-dependent signaling [28].

Regarding the classic RAS family members, and unlike pancreatic, lung and colorectal cancers, activating oncogenic mutations in *KRAS*, *NRAS* and *HRAS* have not often been found in BC [29, 30]. Indeed, the frequency of activating mutations of these three genes together is lower than 1% in genome-wide association studies (GWAS) of BC (www.cbioportal.org). Classic RAS genes, and specially *KRAS*, have been found amplified in their wild type form in BC, including TNBC [30]. However, such amplification of classic RAS members seems to have more an ancillary role in tumorigenesis and metastic behavior than in the generation of BC. In fact, a driver role for mutated or unmutated classic RAS genes has not yet been demonstrated in BC. As for classic RAS genes, the *RRAS2* member of the RAS-related subfamily, also known as TC21, is also infrequently found mutated in BC [31]. Conversely, analysis of a *Rras2* null mouse mutant showed that this GTPase is necessary for correct mammary gland development [32]. Moreover, a polymorphism in the *RRAS2* promoter is associated with an unfavorable tamoxifen treatment outcome [33], and silencing the *RRAS2* gene enhances the tamoxifen sensitivity of a BC cell line [34], indicating *RRAS2* is an important gene in the development and clinical outcome of BC.

Definitive proof for a driver role of *RRAS2* in its unmutated form when overexpressed has been recently generated using a knock-in mouse line at the Rosa26 locus with conditional overexpression of wild-type human *RRAS2* in the mammary gland [35]. All female mice developed breast ductal adenocarcinomas with a median survival of 8 months. Strikingly, BC formation was only detected in breeder females; non-breeders did not develop BC, suggesting that *RRAS2* overexpression is associated with the development of pregnancy-associated BC. *RRAS2* is overexpressed in ~68% of human breast cancers, with this frequency being even higher in TNBC (75%) [35]. In this regard, immunohistochemical and gene expression analysis showed that the ductal adenocarcinomas emerging in the *RRAS2*-overexpressing mouse model corresponded to TNBC and, more precisely, to the mesenchymal stem-like (MSL) subtype. Finally, the increased frequency of a single nucleotide polymorphism in the 3’-untranslated region (UTR) of the *RRAS2* mRNA (rs8570), associated with stronger overexpression of *RRAS2*, in human breast cancer patients compared to the healthy population, strongly links *RRAS2* with human BC.

Here, we have carried out gene expression and interactomics analysis to identify the signaling pathways and the membrane receptors in mouse and human breast cancer cells that could be using R-RAS2 as a signaling effector. We identify R-RAS2 as a key component of CD44 and the CD98hc/LAT1 L-amino acid transporter that mediates metabolism, cytoskeleton remodeling, migration, and metastasis of TNBC cells.

## Materials and methods

### Ethics statement

All animal experiments were carried out at the facilities of the Centro de Biología Molecular Severo Ochoa (Madrid, Spain) in accordance with national and European guidelines (Directive 2010/63/EU). All the procedures were approved by the ethical committee at the CBMSO and by the Regional Government of Madrid (authorization numbers PROEX 384/15 and PROEX 296.7/21).

### Mice

The Rosa26-*RRAS2*^*fl/fl*^ knock-in mouse line was generated by homologous recombination as described in [36], and it contains the WT human *RRAS2* sequence with an HA-tag driven by a CAG promoter, followed by an EGFP sequence downstream of an IRES sequence, and with a LoxP-flanked stop codon at the 5’ end of the construct (Rosa26-*RRAS2*^*fl*/fl^). These Rosa26-*RRAS2*^*fl/fl*^ mice were crossed with different Cre recombinase lines to induce conditional overexpression of this construct by removing the stop codon in specific tissues. We first set out to study systemic *RRAS2* overexpression using the Sox2-Cre transgenic mice kindly provided by Dr César Cobaleda (Centro de Biología Molecular Severo Ochoa -CBMSO, Madrid). As Sox2 is an embryonic stem cell transcription factor, the LoxP-flanked sequence is deleted in all tissues in these mice. By crossing with the WAP-Cre line purchased from the NCI Mouse Repository (Frederick, MD), we next generated a mammary epithelium specific *RRAS2*-overexpressing mouse line, the Rosa26-*RRAS2*^*fl/fl*^xWAP-Cre. In this transgenic strain, the Cre recombinase is expressed under the control of the WAP (whey acidic protein) promoter, specifically in the secretory epithelium of the mammary gland. As such, maximal Cre-mediated recombination is achieved during pregnancy and lactation, although recombined cells are still present after involution and complete remodeling of the gland. Additionally, Rosa26-*RRAS2*^*fl/fl*^xMMTV-Cre mice were also generated by crossing with the MMTV-Cre line F from the NCI Mouse Repository (Frederick, MD). This strain overexpressed *RRAS2* in virgin and lactating mammary gland, and has also been detected in other tissues like salivary gland, seminal vesicle, skin, and cells of the immune system. In vivo xenograft tumor growth assays with CAL-51, MCF7 and BT-549 cells were performed in immunodeficient Rag2^−/−^γc^−/−^ mice purchased from Jackson Laboratories [37]. All the mice were maintained under SPF conditions at the animal facility of the CBMSO, in strict accordance with national and European guidelines.

### RNA sequencing, variant calling and functional enrichment analysis

Thirteen breast tumor samples and nine samples of healthy mammary gland tissue were isolated and immediately prepared for RNAseq. Total RNA extraction was performed with TRIzol® following manufacturer’s protocol. The quality control, library preparation and data processing were carried out at the Centre for Genomic Regulation (CRG, Barcelona, Spain). Samples were sequenced with 50 bp single-end read using a HiSeq 2000 platform (Illumina) after quality control of the samples on a Bioanalyzer Instrument (Agilent). The quality of sequencing data was assessed using FastQC software. The generated libraries were then aligned to the genome reference sequence of *Mus musculus* GRCm39 from Ensembl. The alignment was performed using the Spliced Transcripts Alignment to a Reference (STAR) software and read counting was carried out with the FeatureCounts tool. Differential gene expression analysis was conducted using the DESeq2 package. Ingenuity Pathway Analysis (IPA software, Qiagen) was performed based on differentially expressed genes, selecting the most up-regulated and down-regulated pathways based on the obtained z-scores.

To identify variants from sequence data, variant calling was done using the GATK “RNAseq short variant discovery” pipeline, and the annotation of the variants was done using the SnpEff tool. For a specific mutation to be considered, the read depth (coverage) at the mutation site was required to be at least 20 for each sample. The variants identified through variant calling were subjected to functional enrichment analysis. To perform this analysis, variants were ranked based on several factors: (1) the putative impact according to sequence ontology terms output as defined by the SnpEFF package, (2) the number of tumors harboring the same mutation, and (3) genotype quality. Duplicate genes were removed, retaining only the row with the highest rank value. Subsequently, the ranked genes associated with these variants were used for over-representation analysis using the clusterProfiler R package to link the genes to relevant biological pathways and assess the potential functional impacts of the variants. Specifically, Gene Ontology (GO) annotations and the Kyoto Encyclopedia of Genes and Genomes (KEGG) database were utilized.

### Cell culture

The human BT-549, CAL-51, MCF7, and HEK293T cell lines, and the murine CBM-MBC21 cell line were used in this study. CAL-51, MCF7 and HEK293T cells were cultured in DMEM supplemented with 10% fetal bovine serum (FBS), 2 mM L-glutamine, 100 U/ml penicillin and 100 U/ml streptomycin, while BT-549 cells were maintained in similarly supplemented RPMI. The CBM-MBC21 cell line was generated in the laboratory from a Rosa26-*RRAS2*^fl/fl^ x Wap-Cre breast ductal carcinoma and maintained in DMEM supplemented with 10% FBS, 2 mM L-glutamine, 100 U/ml penicillin, 100 U/ml streptomycin, 20 μM β-mercaptoethanol and 10 mM sodium pyruvate.

### Immunohistochemistry and immunocytochemistry

For immunohistochemical staining, two incubations in xylene for 5 min each were conducted, followed by two times in 100%, 95%, 75%, and 50% ethanol, each for 5 min. Subsequently, samples were treated with methanol + 0.3% H_2_O_2_ for 20 min and washed twice for 5 min with distilled water. Antigen retrieval was carried out by incubating with citrate bufer (100 mM sodium citrate pH 6.0) at boiling temperature for 10 min and, after cooling down, followed by two washes with phosphate-buffered saline (PBS) for 5 min each. Tissue sections were then blocked by incubating with PBS + 1% bovine serum albumin (BSA) at 4 °C for 30 min. Staining with the corresponding primary antibodies was performed overnight in PBS + 3% BSA at 4 °C. The primary antibodies used for immunohistochemistry were Erbb2 (#2165, Cell Signaling, 1:200); PR (Polyclonal, Proteintech, 25,871–1-AP 1:200); ERα (Clon E115, Abcam, ab32063 1:500) and infuenza hemagglutinin epitope (12CA5, Sigma, 1:500). Next, incubation with the corresponding secondary antibody was carried out at 4 °C for 30 min (Polyclonal Biotinylated anti-Rabbit, DAKO E0432, 1:200 and Polyclonal Biotinylated anti-Mouse, Vector Laboratories BA-2000, 1:200). Detection of the antigen–antibody complexes was carried out using the DAB (3,3-Diaminobenzidine chromogen), incubating all samples for the same time. A counterstaining with hematoxylin was performed to stain the nuclei for 10 s, followed by washes in water to remove excess, and rinsing with PBS. Samples were dehydrated by sequential incubations with distilled water for 5 min, followed by two washes of 50%, 75%, 95%, and 100% ethanol for 2 min each, and three 3-min washes with xylene. Samples were mounted using DPX mounting medium. For immunocytochemistry staining, a similar protocol was followed, beginning from the blocking step and performing the incubations with primary and secondary antibodies at the same concentrations. The dehydration of samples after nuclei counterstaining with hematoxylin was not conducted. Samples were analyzed and photographed using a Vertical microscope AxioImager M1 (Zeiss) coupled with DMC6200 camera (Leica) and LAS X software (Leica).

### Antibodies

The antibodies used in this study were directed against: β-Actin (#4967), Akt (#4691), Phospho-Akt (Thr308: #13038), Phospho-Akt (Ser473: #4060), Phospho-PDK1 (Ser241: #3438), Phospho-S6 Ribosomal Protein (Ser235/236: #4858), Phospho-p70 S6 Kinase (Thr389: #97596), 4E-BP1 (#9644), Phospho-4E-BP1 (Thr37/46: #39788), Phospho-FoxO1 (Thr24), Phospho-FoxO3a (Thr32: #9464), Phospho-GSK-3α/β (Ser21/9: #8566), p44/42 MAPK (Erk1/2: #9102), Phospho-p44/42 MAPK (Erk1/2 - Thr202/Tyr204: #4370), Phospho-MEK1/2 (Ser217/221: #9121), Phospho-Raptor (Ser792: #89146), Phospho-Rictor (Thr1135 #3806), mTOR (#2983), Phospho-mTOR (Ser2448: #5536), Epha2 (#6997), Erbb2 (#2165, all from Cell Signaling); PR (Polyclonal, 25,871–1-AP, Proteintech), ERα (E115, Abcam), p53 (PAb240, Abcam), RRas2 (#H00022800-M01, Abnova), Ras (#05-516, Merck); influenza hemagglutinin epitope (12CA5, Sigma); CD44 (#A00052, Boster Bio), CD44 (IM7, BioLegend), hEGFR-BV421 (AY13, BioLegend), CD44-APC (IM7, BD Pharmingen) and CD98 (#MA5-31209, ThermoFisher).

### Real-time quantitative PCR

Samples were resuspended in TRIzol® (Invitrogen), following manufacturer’s protocol. The final pellet was diluted in RNAse-free H_2_O and conserved at -80°C. From the total RNA isolated, cDNA was synthesized with SuperScript III (Invitrogen) using Oligo-dT primers. Quantitative real-time (RT)-PCR was then performed in triplicate using 50 ng cDNA as the template and carrying out the reaction with a SYBR Green PCR Master Mix and gene-specific primers in a CFX384 Touch Real-Time PCR Detection System (Biorad). A specific primer set was used to measure *RRAS2* mRNA expression exclusively in human cells (Fw: 5’-GCAGGACAAGAAGAGTTTGGA-3’; Rv: 5’- TCATTGGGAACTCATCACGA − 3’), and a different primer set was used to measure combined human (*RRAS2*) and mouse (*RRas2*) mRNA expression in mouse samples (Fw: 5’-GAGTTTGGAGCCATGAGAGA-3’; Rv: 5’-CCTTTACTCTGAGAATCTGTCTTTGA-3’). For human samples, the cycle threshold (Ct) values obtained were used to calculate the mRNA levels relative to *PUM1* [38], one of the most stable reference genes to characterize tumors (Fw: 5’-AGTGGGGGACTAGGCGTTAG-3’; Rv: 5’-GTTTTCATCACTGTCTGCATCC-3), and 18 S rRNA expression (Fw: 5’-ATCCATTGGAGGGCAAGTC-3’; Rv: 5’-GCTCCCAAGATCCAACTACG-3’). For mouse samples, the normalizer genes used were C-Terminal Binding Protein 1 (*Ctbp1*), which has been validated as a reliable, tissue-specific reference gene for mouse mammary gland studies [39] (Fw: 5’-GTGCCCTGATGTACCATACCA-3’; Rv: 5’-GCCAATTCGGACGATGATTCTA-3’), and Actin (*Actb*) (Fw: 5’-CTAAGGCCAACCGTGAAAAG-3’; Rv: 5’-ACCAGAGGCATACAGGGACA-3’).

### CRISPR/Cas9 gene editing

*RRAS2* was knocked-out in the MCF7 and BT-549 cell lines using the CRISPR/Cas9 gene editing system according to established guidelines [40]. Target sites were selected by using the CHOPCHOP tool [41], prioritizing to avoid potential off-target effects on other RAS proteins due to their high level of sequence homology [42], and targeted Exon 5 and surrounding region of *RRAS2* sequence. The following primers were generated: Fw: 5’-CACCGGTTTTGCTTACCTGATAACC-3’ and Rv: 5’-AAACGGTTATCAGGTAAGCAAAACC-3’. Cloning was performed using the pSpCas9(BB)-2 A-GFP (PX458) V2.0 vector (Addgene #48138) and the sgRNA oligonucleotides were phosphorylated and annealed to clone them into the PX458 plasmid. The constructs were transformed in competent cells and plated overnight in LB containing Ampicillin (1:1000) at 37 ℃. The plasmid was purified from isolated colonies, and the sgRNA verified by Sanger sequencing. The cell lines were transfected following a liposome-based transfection approach and using the jetPEI® reagent (Polyplus #101–40 N). GFP^+^ clones were selected by cell sorting with a FACSAria Fusion, leaving one GFP^+^ cell per well in a 96-well plate. After growth, western blotting and Sanger sequencing was performed to validate the knock-out.

### Lentivirus production and generation of CAL-51 and CBM-MBC21 *RRAS2* knockdown and BT-549-GFP cell lines

Lentiviral particles were generated in 293T cells cultured in DMEM supplemented with L-glutamine (2 mM) and 10% FBS. The cells were transfected in jetPEI® with 1.7 μg pMD2.G, 3.3 μg psPAX2 and 5 μg of the lentiviral vectors (MISSION® pLKO.1-puro GFP shRNA: Sigma) that target the 5’- CGTGATGAGTTCCCAATGATT-3’ and 5’-CTCAGAGTAAAGGATCGTGAT-3’ sequence for CBM-MBC21 and CAL-51, respectively. Supernatants were collected 48 and 72 h later, and filtered through 0.45 μm filters (Sartorius). The cell lines were transduced with Polybrene (8 μg/ml: Sigma), centrifuging at 900xg and 32 ℃ for 70 min without brakes. Finally, 24 h after infection, the cells were selected with puromycin (6 and 3 μg/ml, respectively). BT-549 control and *RRAS2* KO cells were transduced with lentiviral particles carrying the PHR-SIN-GFP plasmid.

### Flow cytometry

For in vitro proliferation assays, MCF7, BT-549, CBM-MBC21, and CAL-51 cells were seeded in 24-well plates and incubated for 5 days. Cell growth was assessed by measuring viability with 1 μg/ml DAPI and cell counting was performed using CountBright™ Absolute Counting Beads. To minimize the formation of aggregates, 2 mM EDTA was added to the 1% PBS-BSA buffer. Data were acquired using a FACS Canto II cytometer (Becton Dickinson) and analyzed using FlowJo™ software. A minimum of 1000 events from the Counting Beads population were acquired per cytometry tube. This assay was conducted similarly to assess tamoxifen sensitivity of CBM-MBC21, MCF7 and CAL-51 cells, by incubating each cell line at different concentrations for five days. The total cell number according to the acquired counting beads was determined using the following formula: Cell number = Alive cells/Counting beads) * Number of beads per 5 μl.

The effects of *RRAS2* depletion on the cell cycle were evaluated using the APC-BrdU kit from BD Pharmingen™ (552598), following manufacturer’s guidelines. For this analysis, CBM-MBC21 cells were incubated with BrdU for 10 h, whereas BT-549 cells were incubated for 7 h.

To assess viability in Phosflow assays, cells were incubated with Ghost Dye™ Red 780 (TonboBio 13–0865) and fixed for 20 min at 4 ℃ in paraformaldehyde (PFA, 2% final concentration). After washing twice, the cells were permeabilized for 30 min with 90% methanol on ice and intracellular labeling was then performed overnight (O/N) at 4 ℃ with specific antibodies, using Alexa 647 Donkey anti-Rabbit IgG as the secondary antibody for 40 min at RT.

To evaluate the mTORC1 response to Leu, Ile, Val, and Gln deprivation, amino acid availability was specifically altered but not other cell culture parameters. BT-549 and CBM-MBC21 cells were seeded the prior day in RPMI/DMEM containing 2% FBS to ensure cell survival. On the day of the assay, serum was removed, and the medium was replaced with Leu-, Ile-, Val-, and Gln-free media for 1.5 h. Subsequently, the medium was replaced with media containing a full complement of amino acids but no serum. Cells were fixed with 2% PFA and stained at different time points (0, 90, 120, 240 and 360 min) for phospho-S6 Ribosomal Protein (Ser235/236) detection by flow cytometry, as described above. Additionally, cells were also lysed for Western blotting at 0-, 90-, 120-, and 240-minutes post-re-addition of amino acids. As control, Rapamycin (Stemcell technologies) was used at 50nM.

### Orthotopic tumor growth assay

CAL-51 control or CAL-51 shRRAS2 (short hairpin RNA targeting *RRAS2* mRNA) (4 × 10^6^ cells); MCF7 control or MCF7-RRAS2 KO and BT-549 control or BT-549-RRAS2 KO cells (5 × 10^6^ cells) were orthotopically injected in a total volume of 100 μL of PBS/Matrigel. The injection was performed into the left fat pad of the fifth pair of 10-week-old *Rag2*^−/−^γc^−/−^ female mice. For CBM-MBC21 control or CBM-MBC21 shRRAS2 cells (4 × 10⁶ cells), injections were carried out in wild-type nulliparous C57BL/6 females. To study metastatic processes while retarding the growth of the primary tumor, a lower number of cells were injected (50,000 BT-549 cells and 250,000 CBM-MBC21 cells). For cell injection, surgery was conducted following described procedures [43]. A small incision was made between the fourth right nipple and the midline using a scalpel, exposing the right mammary fat pad from the fourth pair for injection of tumor cells. After cell inoculation, the incisions were sutured, and oral analgesics were administered to alleviate pain. Body weight and tumor growth were monitored regularly, ensuring compliance with ethical guidelines to minimize discomfort to the animals. The volume of mammary tumors was calculated using the formula V = (W2 × L)/2 for caliper measurements (V = volume, W = width, L = length). The endpoint of the experiment was reached when any of the tumors exceeded the volume limit set by ethical guidelines. At this point, tumors were isolated and weighed, and organs commonly targeted by breast cancer metastasis, such as the lungs and liver, were harvested to check for metastatic BT-549 or CBM-MBC21 cells. These organs were examined for macroscopic nodules, by immunohistochemistry and by flow cytometry.

### Interactome analysis by mass spectrometry

A freshly-isolated breast tumor emerging in a Rosa26-RRAS2^fl/fl^ x Wap-Cre female breeder and weighing 1.5 g was fragmented in small pieces using a scalpel and passed through a cell strainer. A suspension of 131 × 10^6^ cells in PBS was obtained and diluted to a concentration of 10 × 106/mL. The plasma membrane proteins were biotin-labeled by incubation for 1 h on ice with Ez-Link SULFO-NHS-LC-Biotin (ThermoFisher) at a concentration of 1 mg/mL, and the cells were then concentrated by centrifugation and lysed in 0.5% Brij96 lysis buffer containing protease and phosphatase inhibitors: 0.5% Brij96, 140 mM NaCl, 20 mM Tris-HCl (pH 7.8), 10 mM iodoacetamide, 1 mM phenylmethylsulfonyl fluoride (PMSF), leupeptin (1 mg/ml), aprotinin (1 mg/ml), 1 mM sodium orthovanadate and 20 mM sodium fluoride. Co-immunoprecipitation with anti-Hag (12CA5, Sigma) or isotype control (Mouse IgG2b kappa Isotype Control: ThermoFisher) was performed with the Pierce™ Co-IP Kit (ThermoFisher) following the manufacturer’s instructions. An additional purification step was carried out incubating the Co-IP eluate with Streptavidin Sepharose® Beads (Cell Signaling) in order to isolate the membrane components.

Protein complexes were recovered with SDS gel-loading buffer and the entire sample was run on SDS–polyacrylamide gel electrophoresis (PAGE) 12% gels until all proteins had just entered into the gel, then stained with QuickCoomassie and digested with trypsin using a standard in-gel sample digestion method. The tryptic peptides were eluted and analyzed using liquid nanochromatography coupled with mass spectrometry on an Orbitrap Exploris OE240 system at the Proteomics Facility of the National Center for Biotechnology. The spectra obtained were used to search a target-decoy database of approximately 111,000 *Mus musculus* entries, including more than 100 entries corresponding to typical laboratory contaminants. The results were filtered, retaining only those proteins with at least one unique (specific) peptide and with a Mascot score equal to or higher than 31, the value automatically calculated as a cut-off value to separate statistically significant identifications (*p* < 0.05) from non-significant ones. After excluding common laboratory contaminants, the resulting list of 553 proteins was analyzed for over-representation using the clusterProfiler R package. This analysis linked the genes to relevant biological pathways and evaluated the potential functional impacts of the variants, using annotations from the KEGG database.

### Co-immunoprecipitation and Western blotting

To analyze whole-cell lysates in Western blots, the cells were lysed in Brij96 lysis buffer with protease and phosphatase inhibitors (see above), resolving the lysates by SDS-PAGE and transferring the proteins to nitrocellulose membranes using a wet transfer procedure (Biorad). The membranes were blocked for 1 h in 5% BSA (Sigma) in TBS-T (25mM Tris-HCl [pH 8.0], 150mM NaCl, 0.1% Tween-20) and then incubated overnight at 4 °C with the appropriate primary antibodies diluted in blocking buffer. After three washes with TBS-T, the membrane was incubated for 45 min at RT with the secondary antibody (1:30,000 dilution: Jackson Immunoresearch) and antibody binding was detected by standard chemoluminescence with a Kodak X-OMAT 2000 Processor. Co-immunoprecipitation assays were performed on human BT-549 BC cells transfected with an Hag-tagged EGFP-*RRAS2* vector and on mouse CBM-MBC21 cells. The cells were lysed for 30 min on ice in 0.5% Brij96 lysis buffer with protease and phosphatase inhibitors and co-immunoprecipitation was carried out by incubating the cytosolic fraction O/N at 4 °C under continuous rotation with 10 μg of anti-Hag (12CA5. Sigma) or an IgG2b isotype control (eBMG2b, ThermoFisher) previously bound to Protein G Sepharose® beads (Sigma). The beads were recovered and washed five times with Brij96 lysis buffer, and finally resuspended in 40 μL of SDS gel-loading buffer to perform SDS-PAGE under non-reducing or reducing conditions. The immunoprecipitated proteins were probed with antibodies against Epha2, CD44, CD98 and Hag.

### CD44 isoform characterization

Characterization of the mouse CD44 isoforms in Rosa26-*RRAS2*^fl/fl^ x Wap-Cre breast tumors was carried out with cDNA synthesized from tumor RNA. PCR was performed using the Fw 5’-AGGAAATGTGGTAATTCCGAGGA-3’ (aligned to CD44 exon 3) and Rv 5’-CAGATTCCGGGTCTCGTCAG-3’ (aligned to exon 19) primers, and GoTaq® Flexi DNA Polymerase. The PCR products amplified were run on a 1% agarose gel and purified with QIAquick Gel Extraction Kit (Qiagen). The gel-purified PCR product was cloned into pCR 2.1-TOPO cloning vector (Invitrogen) for subsequent sequencing. Ten colonies from each cloning were sequenced.

### Measurement of cellular oxygen consumption and extracellular acidification

The oxygen consumption rate (OCR) and extracellular acidification rate (ECAR) were measured using a Seahorse XFe96 Analyzer (Agilent). On the previous day, CBM-MBC21 control and shRRAS2 cells (15,000–20,000 cells per well, six wells per treatment for each independent experiment) were plated. The cells were then preincubated for 1 h at 37 °C with Seahorse XF DMEM medium supplemented with 25 mM glucose, 1 mM pyruvate, and 2 mM glutamine in a CO_2_-free incubator. OCR measurements were taken following successive injections of 2 μM oligomycin, 1.5 μM FCCP, and 1 μM antimycin A/rotenone. After the assay, cells were stained with 1 μg/ml Hoechst for cell counting and normalization.

In addition, mitochondrial fuel dependency, flexibility, and capacity in CBM-MBC21 cells were assessed using the MitoFuel Flex test. For this assay, 20,000 CBM-MBC21 control and shRRAS2 cells were seeded in sextuplicate and preincubated as described above. Fuel oxidation capacity was measured by sequential injections of 2 μM UK5099, 3 μM BPTES, and 4 μM Etomoxir, in different combinations to assess the cells’ dependency, capacity and flexibility on glucose, glutamine, and fatty acids.

### Immunofluorescence

CBM-MBC21 cells were seeded onto sterile coverslips in 24-well plates and incubated overnight. Cells were then fixed with 2% PFA for 20 min on ice, permeabilized with 0.1% Triton X-100 in PBS for 10 min at RT, and blocked with PBS 2% BSA for 45 min at RT. Following blocking, cells were incubated overnight at 4 °C with a mouse monoclonal antibody against R-RAS2 (targeting the Hag tag, 12CA5 clone, Sigma) and rabbit monoclonal antibodies against CD44 or CD98 (Boster Bio and ThermoFisher, respectively). After washing, coverslips were incubated for 1 h at RT with Alexa Fluor 555 donkey anti-mouse IgG and Alexa Fluor 647 donkey anti-rabbit IgG. Cells were then washed twice, stained with DAPI (1 μg/ml, Merck) for 5 min at RT, mounted using ProLong™ Glass Antifade Mountant (ThermoFisher), and analyzed using an LSM710 confocal microscope (Zeiss) with a 63x Plan-Apochromat objective. Colocalization analysis was performed in Fiji using a one-to-one pixel matching approach on individual z-stacks. Regions of interest (ROIs) on the plasma membrane were selected, and colocalization was assessed using the Colocalization Finder plugin (Philippe Carl, Université de Strasbourg).

To investigate the behavior of CBM-MBC21 cells in the presence of CD44’s ligand, hyaluronan, sterile coverslips were first treated with 100 μg/ml poly-L-lysine and 2 mg/ml hyaluronan overnight at 4 °C. The following day, cells were seeded and allowed to spread on the coverslips for 4 h. In some cases, cells were pre-incubated with the CD44-blocking antibody IM7 for 30 min on ice. Subsequently, cells were fixed and stained for R-RAS2 using the anti-Hag antibody, for phosphorylated AKT at Ser473 (p-AKT S473, #4060), and for F-actin using Phalloidin Alexa Fluor 555. Cell area and protrusion length were quantified using a custom Fiji macro developed by the CBMSO Advanced Microscopy Facility, which determined the center of mass for each cell and measured the length of individual protrusions (Extended Data Table 8).

Confocal microscopy was also used to study the activation levels of R-RAS2 as part of a Förster Resonance Energy Transfer (FRET) biosensor system. These biosensors are widely used in live-cell imaging to monitor molecular interactions or signaling events by measuring FRET efficiency between the donor fluorophore (Cerulean) and the acceptor fluorophore (Venus). Specifically, our biosensor measures the association of R-RAS2 with the Ras Binding Domain (RBD) of the p110δ subunit (P110 RBD), one of its effectors. To assess this, CBM-MBC21 cells were transfected with 3 μg of the biosensor plasmid using JetPEI® transfection reagent. The following day, cells were seeded onto hyaluronan-treated coverslips. After 4 h of incubation, cells were fixed with 2% PFA in PBS for 20 min on ice and analyzed using an LSM710 confocal microscope with a 63x Plan-Apochromat objective. Activation levels of R-RAS2 in various cellular regions were studied using a custom ImageJ macro, which quantified the FRET ratio (FRET signal normalized to Cerulean signal) across subcellular regions, distinguishing between protruding and non-protruding areas (Extended Data Table 8).

### In vitro cell migration and invasion assays

Cell migration and invasion assays were performed following Corning Life Sciences protocol guidelines. All experiments were carried out using 8 μm pore diameter polycarbonate membranes packaged in p24-well plates (Corning #3422). For the invasion assays, membranes were previously coated with 100 μl of Corning Matrigel® basement membrane matrix at 450 μg/ml. The Matrigel mixture consists of approximately 60% laminin, 30% collagen IV, 8% entactin and 2–3% heparin sulfate proteoglycan perlecan. The coated membranes were allowed to polymerize for 1 h at 37 °C. In some experiments, 2 mg/ml hyaluronan was added to the Matrigel mixture in the upper chamber to assess its effect on cell invasiveness. Additionally, cells were incubated with 5 μg/ml anti-CD44 antibody (IM7 clone) in some cases to evaluate the effects of blocking CD44 in our system. Subsequently, CBM-MBC21 control, CBM-MBC21 shRRAS2, BT-549 control and BT-549 RRAS2 KO were seeded in transwell membranes at a density of 75.000 cells/well in DMEM or RPMI without FBS. In the lower chamber, media with 20% FBS was used as a chemoattractant. After overnight incubation, any cells that had not migrated to the lower chamber were gently removed from the upper chamber using moistened cotton swabs. The upper chamber was then rinsed with PBS. The cells that had migrated to the lower chamber were fixed in 100% ice-cold methanol for 20 min on ice. Then, these cells were stained with 0.2% Crystal Violet for 10 min at RT. After staining, the cells were washed with H2O as required. Transwell membranes were removed from their supports and prepared for visualization with one drop of Permount™ mounting medium (Fisher scientific) per membrane. Images of attached migrating and invasive cells were taken with an AxioImager M1 upright microscope coupled to a DMC6200 camera and equipped with LAS X software. For cell quantification, the ImageJ software was utilized. To determine the number of migrating and invasive cancer cells per mm², various parameters were considered, including the membrane’s diameter (6.5 mm), membrane area (33.18 mm²), and the area covered by each picture at a 10x magnification (0.207 mm²; 576 × 360 μm).

### Statistical analysis

Statistical parameters, including the exact *n* value, and the mean ± S.D. or SEM, are described in the Figures and Figure legends. Non-parametric Wilcoxon Mann–Whitney U tests, and parametric Student’s T-tests, and One-way and Two-way ANOVA tests, were used to assess the significance of the mean differences, as indicated. Experiments were repeated independently as described in the figure legends. The number of mice used for comparison was calculated from the preliminary experiments with the aim of generating significant data to give an alpha = 0.05 and a standard deviation of about 0.3 when a two-sided T-test was used. The different deviation of the control and test groups suggested the use of different numbers of each animal type for the definitive experiments. Western blotting was performed at least twice with similar results and one representative image is shown in the Figures. All the data were analyzed using the GraphPad Prism 9 software.

## Results

### Convergent patterns of recurrent somatic mutations in independent *RRAS2*-driven breast tumors in mice

**Fig. 1:**
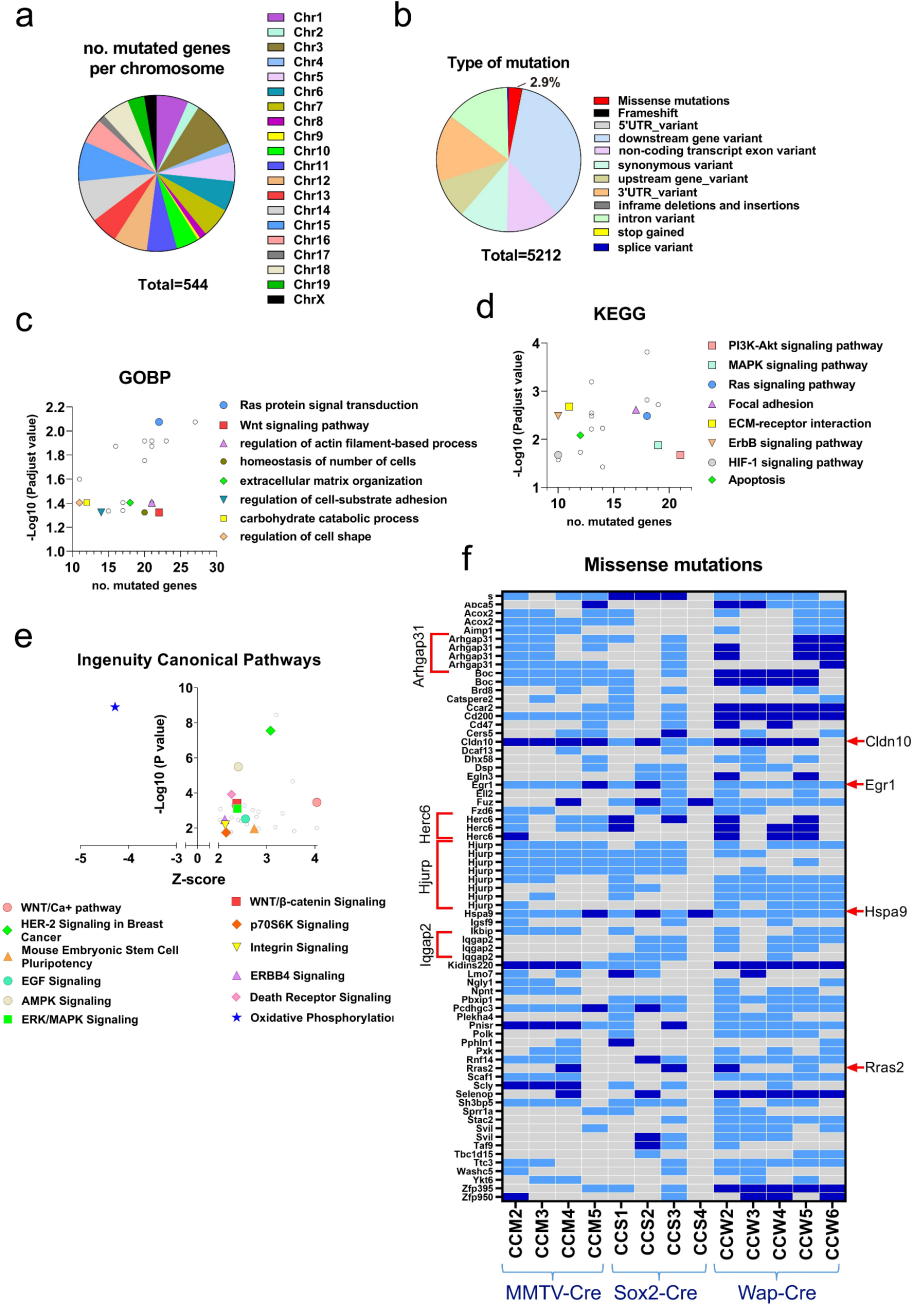
Breast tumors that emerge in *RRAS2 *-overexpressing mice are associated with the mutation of genes known to be important for human breast cancer. **a**, Pie chart of the chromosomal distribution of all genes (*n* = 544) found mutated in breast tumors from 13 *RRAS2*-overexpressing mice (4 from R26-*RRAS2*^fl/fl^ x MMTV-Cre; 4 from R26-*RRAS2*^fl/fl^ x Sox2-Cre; 5 from R26-*RRAS2*^fl/fl^ x Wap-Cre). Full data is provided in Extended Data Table 1. **b**, Pie chart of the 5,212 mutations found in the 544 genes classified according to the transcript biotype (Extended Data Table 1). **c**, Biological processes found to be significantly altered after Gene Ontology Biological Process (GOBP) analysis of the 544 mutated genes. The x-axis shows the number of genes in the pathway found mutated and the y-axis the–Log10 of the Padjusted value. Full data is provided in Extended Data Table 2. **d**, Biological processes and pathways found to be significantly altered after an enrichment analysis of the 544 mutated genes using annotations from the KEGG database. xy plot is as in Fig. 1c. Full data is provided in Extended Data Table 2. **e**, Ingenuity Canonical Pathways with *p* < 0.05 of RNAseq data of 13 tumors samples compared to 9 healthy tissue (full data in Extended Data Table 4). The plot highlights a selected list of pathways activated or inhibited in breast cancer tumors: a positive Z score means upregulation in tumors; a negative Z score, downregulation. **f**, Heatmap of the missense mutations found in at least one tumor from each type of transgenic mice (MMTV-Cre, Sox2-Cre and Wap-Cre). The code given to each tumor is indicated at the bottom of the panel. Dark blue color indicates the mutation being found in 100% of the mRNA sequences; light blue indicates mutations found in approximately 50% of the sequences and grey color indicates no mutation. Some genes were mutated at more than one position in several tumors (e.g. *Hjurp*) and this is indicated with brackets on the left. Red arrows on the right highlight those genes that are found mutated in at least 8 tumors. The endogenous *Rras2* gene (red type) is mutated in four independent tumors. Full data is provided in Extended Data Table 5

To gain insight into the alterations induced by *RRAS2* overexpression in breast tumors, we performed RNAseq analysis on thirteen independent tumors that emerged in eight Rosa26-RRAS2^fl/fl^ mice (two MMTV-Cre+, four Sox2-Cre+, two Wap-Cre+; see Extended Data Table 1) that had been through one or more pregnancies, and nine samples of normal mammary gland tissue (two non-transgenic nulliparous females, three non-transgenic breeders, two Cre-Rosa26-RRAS2^fl/fl^ nulliparous females, and two Cre-Rosa26-RRAS2^fl/fl^ pregnant females) [35]. In the RNAseq data, we found a considerable number of somatic mutations in numerous transcribed genes. We considered mutations to be valid when at least 20 RNA reads per sample contained the same nucleotide change at the same position in the same gene. In the total thirteen tumor samples analyzed, we found 5,212 mutations (Extended Data Table 1) in 544 genes distributed throughout most of the autosomal and X chromosomes (Fig. 1a). The majority of mutations (1,826) were downstream gene variants, whereas only 154 mutations (2.9%) corresponded to missense mutations (Fig. 1b).

**Table 1:** Genes found with missense mutations in at least five independent tumors

Gene	Description*	Protein function*	Cancer prognostics summary*
Aaas	Aladin WD repeat nucleoporin	nuclear pore complex, centromeric	unfavorable marker in renal cancer
Acox2	Acyl-CoA oxidase 2	degradation of fatty acids	associated to **breast cancer** survival
Arhgap31	Rho GTPase activating protein 31	GAP for Rac1 and Cdc42	required for **ErbB-2** induced cell motility and invasion
Boc	BOC cell adhesion associated, oncogene regulated	component of a cell-surface receptor complex that mediates cell-cell interactions	unfavorable marker in urothelial cancer. Favorable in head and neck cancer
Cd200	CD200	plasma membrane receptor	immunosupression of anti-tumor activity
Cldn10	Claudin 10	component of tight junctions in plasma membrane	SNP associated with increased risk of **breast cancer**
Egr1	Early growth response 1	tumor suppressor, activates expression p53 and TGFbeta1	prognostic value in **breast cancer** progression
Fzd6	Frizzled class receptor 6	receptor for Wnt proteins	frequently amplified in **breast cancer**
Herc6	HECT and RLD domain containing E3 ubiquitin protein ligase family member 6	E3 ubiquitin ligase	favorable in renal cancer
Hjurp	Holliday junction recognition protein	maintains histone H3 CENPA at centromers	prognostic marker for **luminal A breast cancers**
Hspa9	Heat shock protein family A (Hsp70) member 9	mitochondrial chaperone	overexpression related to **metastatic breast cancer**
Iqgap2	IQ motif containing GTPase activating protein	binds to activated CDC42 and RAC1	favorable in liver and renal cancer
Kidins220	Kinase D interacting substrate 220	promotes Rap1-dependent ERK activation	favorable in lung and renal cancer
Pcdhgc3	Protocadherin gamma subfamily C, 3	potential calcium-dependent cell-adhesion protein.	unfavorable marker in urothelial, ovarian and renal cancer
Pnisr	PNN interacting serine and arginine rich protein	nuclear speckles	unfavorable marker in renal cancer, favorable in pancreas and urothelial cancers
Rnf14	Ring finger protein 14	E3 ubiquitin ligase. coactivator for progesterone-dependent transcription	unfavorable for thyroid cancer, favorable for renal cancer
Rras2	RAS related 2	G23S mutation found in one invasive lobular breast cancer	unfavorable marker in pancreatic, and head and neck cancers
Scaf1	SR-related CTD associated factor 1	pre-mRNA splicing	unfavorable in colorectal cancer
Sh3bp5	SH3 domain binding protein 5	guanine nucleotide exchange factor for RAB11A and RAB25	unfavorable marker in renal cancer
Svil	Supervillin	high-affinity link between the actin cytoskeleton and the membrane	unfavorable in urothelial cancer

*Gene descriptions, protein function and association with cancer have been retrieved from The Human Protein Atlas: https://www.proteinatlas.org

Using the list of genes bearing mutations, we carried out a functional enrichment analysis to understand the impact of these mutations on biological processes and signaling pathways. The analysis using the Gene Ontology Biological Processes database (GOBP) showed a pattern of mutations in components of the RAS protein signal transduction pathway, which was expected given that the 13 breast tumors emerged in mammary glands overexpressing *RRAS2* (Fig. 1c and Extended Data Table 2). This impact on the RAS signaling pathway was also highlighted after the analysis of mutated genes using the Kyoto Encyclopedia of Genes and Genomes database (KEGG, Fig. 1d and Extended Data Table 2). The finding of an effect of gene mutations on the KEGG PI3K-Akt and MAPK signaling pathways is consistent with *RRAS2* overexpression (Fig. 1d). Additionally, the GOBP analysis suggested an impact of gene mutations on extracellular matrix (ECM) organization and regulation of cell-substrate adhesion, as well as an effect on the regulation of actin filament-based processes (Fig. 1c). These effects on genes that determine interaction with the ECM (ECM-receptor interaction) and actin cytoskeleton (focal adhesions) were also found after KEGG analysis (Fig. 1d). Interestingly, the GOBP analysis also showed an impact of the mutations on the Wnt signaling pathway (Fig. 1c). This effect on the Wnt pathway was also found after Ingenuity Pathway Analysis (IPA) of mRNA expression levels when all transcripts were considered for analysis regardless of their mutational status (Fig. 1e and Extended Data Tables 3 and 4). We would like to highlight that the IPA analysis suggested the activation of both the canonical Wnt/β-catenin and non-canonical Wnt/Ca + + pathways, together with the fingerprint of mouse embryonic stem cell pluripotency, since the Wnt pathways have an important role in TNBC and cancer cell stemness [44] (Fig. 1e).

When we restricted our analysis to the 101 genes bearing missense mutations, we found that some of them were repeated at the same nucleotide position in different independent tumors (Extended Data Table 5). A summary of the genes with protein-altering mutations found in at least one tumor sample per type of knock-in mouse line (Sox2-Cre, MMTV-Cre, and Wap-Cre) is shown in Fig. 1f. These rates suggested that most cells in the tumors bear the mutations and that, assuming diploidy, they affected either one or both of the alleles. Interestingly, some genes like *Hjurp*, *Arhgap31*, *Herc6*, and *Iqgap2* were mutated at more than one position and in different tumors. Others were mutated in the majority of the independent tumors at the same position. For instance, *Hspa9* was mutated in all thirteen tumors; *Egr1* and *Cldn10* were mutated in twelve (Fig. 1f). These findings suggest the existence of a strong selective pressure for the mutation of a selected group of genes at the same positions in our models of *RRAS2*-induced BC development. Some of the genes found with somatic mutations that alter the protein sequence in at least three independent tumors (Fig. 1f) have been previously associated with cancer and some with BC (Table 1 and Supl. Fig. S1a).

Notably, Rosa26-RRAS2^fl/fl^ Cre + tumors overexpress wild-type human *RRAS2*, but in addition, 4 of the 13 tumors sequenced bore the G23S somatic mutation (equivalent to the G12S mutation in *KRAS*) in the endogenous murine *Rras2* gene (Fig. 1f; Table 1). The *Rras2* G23S mutations were all found in tumors from parous female mice that were not either pregnant or lactating at the time of tumor finding. Although activating mutations in *RRAS2* are seldom found in human BC (they are found in 3 of 8,906 tumor samples; cBioportal.org), the somatic mutation G23S in the endogenous *Rras2* locus suggests another step in BC evolution on top of wild-type *RRAS2* overexpression.

In line with the analysis of GOBP processes and KEGG pathways on total gene mutations (Fig. 1c and d), it is striking the number of genes encoding for proteins related to cell-cell interactions and cell motility that are found bearing missense mutations (Fig. 1f). These include: *Arhgap31*, *Boc*, *Catspere2*, *Cd47*, *Cldn10*, *Dsp*, *Fuz*, *Igsf9*, *Iqgap2*, *Npnt*, *Pcdhgc3*, *Pphln1*, *Sprr1a*, and *Svil*. Subsequently, we studied the association in co-occurrence or mutual exclusivity within 994 samples of human breast invasive carcinoma in The Cancer Genome Atlas (TCGA) of the latter list of genes. We found 13 significant associations in co-occurrence (Suppl. Fig. S1b). Among those, high *RRAS2* mRNA expression associated with alterations in *Svil* (supervillin) and *Cd47* (CD47). CD47 mediates cell-cell interactions, modulates integrin signaling and has a role in preventing phagocytosis of tumors cells by cells of the immune system [45]. Supervillin is an actin- and membrane-associated protein involved in cell spreading, lamellipodia extension, actin filament assembly, and regulation of focal adhesions [46].

Overall, the data shown here suggest the existence of a convergent process of mutagenesis and mutant selection that has taken place in a parallel manner in the thirteen tumor samples analyzed that alter cell stemness, the actin cytoskeleton, cell-cell contact and interactions with the ECM.

### *RRAS2* dependency for tumorigenesis of murine and human breast cancer cells

**Fig. 2:**
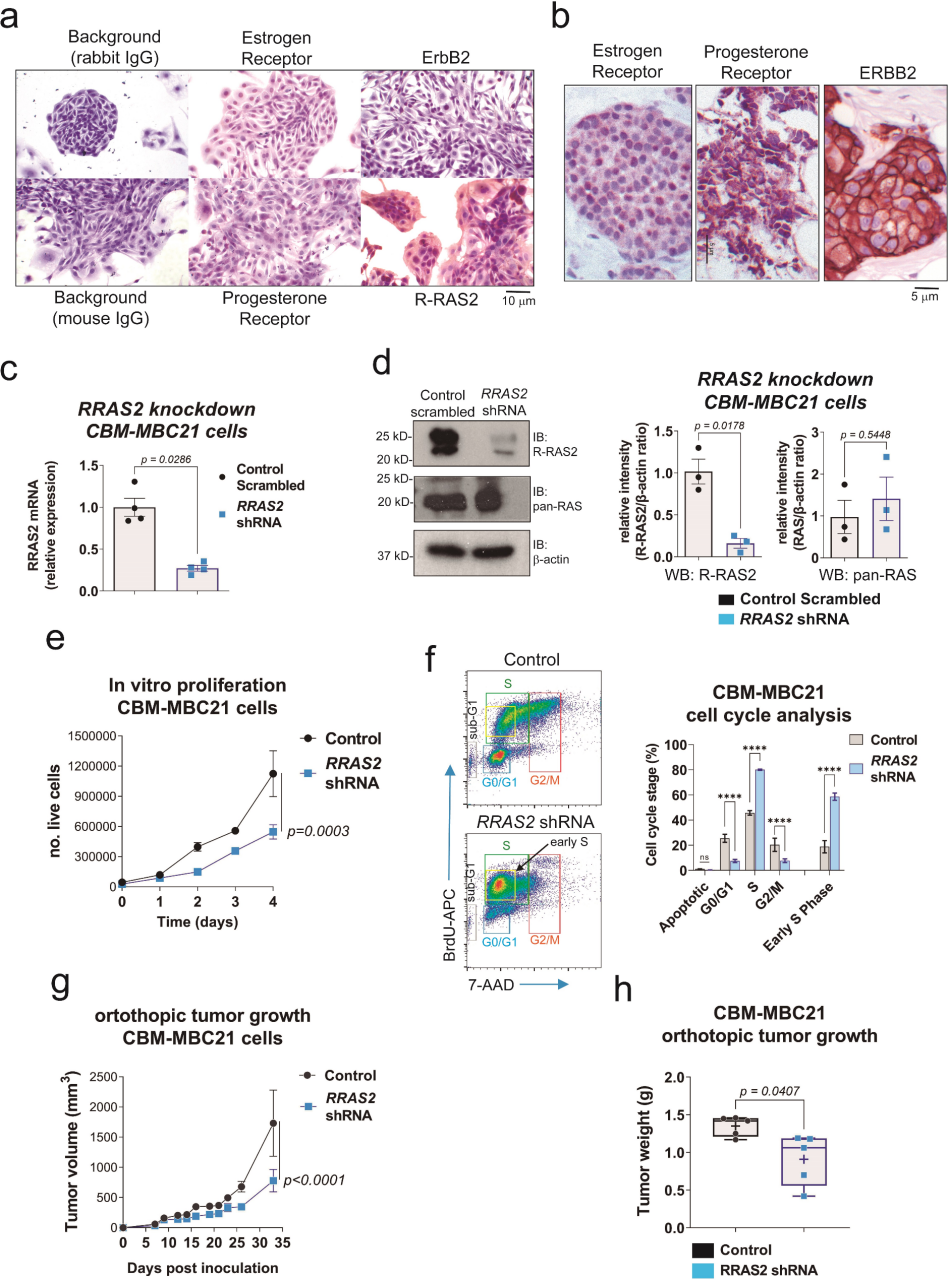
Generation and RRAS2-dependency of a murine TNBC cell line derived from a spontaneous tumor growing in Rosa26-*RRAS2*^fl/fl^xWap-Cre mice. **a**, Immunohistochemical characterization of the CBM-MBC21 murine breast cancer cell line. Hematoxilin and eosin counterstains of immunoperoxidase stainings carried out with the indicated rabbit (estrogen receptor; ErbB2) and mouse (progesterone receptor; R-RAS2) antibodies. Control stainings with unspecific antibodies of mouse and rabbit origin were carried out in parallel. **b**, Immunoperoxidase staining of tissue paraffin sections from human breast cancer samples positive for ERα (luminal A), PR (luminal A), or ERBB2 (HER2-enriched) were stained with the same antibodies and used as positive controls of Fig. 2a stainings. ERα staining is clearly concentrated in the nuclei of the positive cells, ERBB2 staining produced a plasma membrane staining, and PR staining resulted in a mixed, mostly cytoplasmic pattern. **c**, A *RRAS2* knockdown cell line derived from CBM-MBC21 cells was derived using a shRNA construct. The bar plot shows the mean ± s.e.m. of *RRAS2* mRNA expression in the original and the knockdown CBM-MBC21 cell lines determined by RT-qPCR. Significance was assessed using a non-parametric Mann-Whitney test. **d**, Western blot analysis of R-RAS2 protein expression in CBM-MBC21 and knockdown cells. Immunoblot (IB) loading and specificity controls were carried out by incubation with an anti-actin and an anti-pan-RAS (classical) antibodies. The bar plots to the right shows the quantification of the R-RAS2 band referred to the actin band, as well as a quantification of total classical RAS protein detected with the pan-RAS antibody and referred to the actin band. Data were generated by densitometry of western blots run in triplicate. Data is represented as the mean ± s.e.m. Significance was assessed using a non-parametric Mann-Whitney test. **e**, In vitro proliferation of *RRAS2* knockdown CBM-MBC21 and control (CBM-MBC21 transduced with a scrambled shRNA construct) cells was assessed by counting the number of live cells at the indicated time points. The line plot shows the the mean ± s.e.m. of triplicates. Significance was assessed using a two-way ANOVA test. **f**, Analysis of the cell cycle in control and *RRAS2* knockdown CBM-MBC21 cells after a 10-hour incubation with BrdU and double staining with anti-BrdU-APC antibody and 7-AAD. The positions of cell populations at different stages of the cell cycle are indicated with colored rectangles. The presence of a cell population with high incorporation of BrdU but little amplification of the DNA (low 7-AAD) is indicated with a yellow square as a population in early S-phase. The bar plot to the right shows the quantification of cells at different stages of the cell cycle as the mean ± S.D. of triplicates. Significance was assessed using a two-way ANOVA test. ****, *p* < 0.0001. **g**, In vivo proliferation in orthotopic (breast) location was assessed after inoculating 5 × 10^6^ cells into the left inguinal mammary gland of C57BL/6 female mice and by measuring the volume of the tumor protrusion at skin level at the indicated time points. Datapoints show the mean ± s.e.m. for *n* = 5 implanted mice per cell line. Significance was assessed using a two-way ANOVA test. **h**, Tumor weight was measured at day of termination (day 33). A box and whiskers plot showing the median, the mean (+) and all datapoints is displayed. Significance was assessed using an unpaired t-test with Welch’s correction

R26-RRAS2^fl/fl^ x Wap-Cre female breeder mice, along with the other two Cre-expressing transgenic mice, develop breast cancer [35]. Unlike established cell lines that have randomly generated from breast tumor samples, the tumors originate in R26-RRAS2^fl/fl^ x Wap-Cre mice as a consequence of overexpression of wild type *RRAS2*. Therefore, they offer a unique opportunity to study the role of this GTPase in BC cell biology. To this aim, we established a cell line from a primary tumor isolated from an R26-RRAS2^fl/fl^ x Wap-Cre female breeder mouse. The cell line, termed CBM-MBC21, has been maintained in vitro for 5 passages. Immunohistochemical analysis of the cell line showed that it overexpresses the R-RAS2 protein and, that like the freshly isolated mouse tumors [35], it is negative or low for estrogen receptor, progesterone receptor, and HER2 (*ErbB2*), and therefore can be considered triple-negative, in comparison with human tumor controls (Fig. 2a and b). Indeed, unlike the luminal human MCF7 cell line, CBM-MBC21 was resistant to tamoxifen treatment, showing estrogen receptor independence (Supl. Fig. S2). The CBM-MBC21 cell line exhibits a spindle-like shape in culture (Fig. 2a), consequent with the classification of the parental TNBC tumors generated in R26-RRAS2^fl/fl^ x Wap-Cre female breeder mice as mesenchymal stem-like (MSL) [4, 5, 35].

To study the effect of R-RAS2 depletion on CBM-MBC21 cell behavior, we first attempted to generate a total *RRAS2* knockout using the CRISPR/Cas9 method, but did not succeed in isolating such cell clones. We therefore aimed to generate knockdown clones using a shRNA construct previously tested to be effective in other cell lines [36]. We generated a CBM-MBC21 derivative that had reduced the expression of the human *RRAS2* mRNA by 4-fold compared with the control line transduced with a scrambled shRNA construct (Fig. 2c). Expression of the human R-RAS2 protein expression was also reduced by 4-fold, whereas classical RAS proteins, which run with slightly faster mobility in SDS-PAGE, were not reduced (Fig. 2d).

The effect of R-RAS2 depletion on the proliferation rate of CBM-MBC21 cells was first studied in vitro. *RRAS2*-knockdown cells proliferated at a 50% lower rate than the control cell line (Fig. 2e). Interestingly, cell cycle analysis showed that slower proliferation rate of *RRAS2*-knockdown CBM-MBC21 cells was not due to an accumulation in G1 or G2/M phases, or to increased apoptosis, but to a partial arrest at an early S phase (Fig. 2f). This highlights a need for high *RRAS2* expression to promote DNA replication beyond initiation of DNA synthesis.

The effect of R-RAS2 depletion on tumor growth in vivo at the orthotopic location was also investigated by inoculating the control and knockdown cells into the left inguinal mammary gland of non-breeder 8-week-old C57BL/6 female mice. Tumor growth was monitored on different times post-inoculation by measuring the volume of the external protrusion. This showed that R-RAS2 knockdown cells grew at a slower pace than the controls, resulting in a ~60% volume reduction on the day of sacrifice (day 33, Fig. 2g). Breast tumors were extracted after necropsy and weighed. The results showed a significantly lower mean and median tumor weight in the knockdown group compared to controls (Fig. 2h).

Altogether, the results in Fig. 2 show that high expression of human *RRAS2* is required to maintain the oncogenic properties of a mouse BC cell line derived from a knock-in mouse line overexpressing human unmutated *RRAS2*. These findings demonstrate that *RRAS2* overexpression not only drives the generation of BC in mice but that it is also necessary to maintain the malignant phenotype, bringing the research full circle.

Although we had previously shown that knockdown of *RRAS2* expression in the human triple negative breast cancer cell line MDA-MB231 was required for tumor generation in experiments of xenograft [47], we aimed to reinforce those data by incorporating three additional human cancer cell lines of different types. Besides, MDA-MB231 cells bear an activating G13D mutation in *KRAS* and this could alter the dependency on R-RAS2 expression. We chose two triple negative cell lines (BT-549 and CAL-51) and one ER positive derived from a luminal A tumor (MCF7) (Suppl. Fig. S3a). None of the three cell lines bears an activating mutation in any of the classical *RAS* genes, although CAL-51 bears an activating mutation in *RRAS2*; the other two overexpress wild type *RRAS2* by ~8-fold (Suppl. Fig. S3a). Unlike for MDA-MB231, we tried to generate full *RRAS2* knockout cell lines rather than knockdown ones. We succeeded for BT-549 and MCF7 but not for CAL-51. For the latter, we generated a *RRAS2* knockdown cell line using our shRNA-expressing lentiviral vector [36]. Western blot analysis confirmed total absence of R-RAS2 expression in the knockout BT-549 and MCF7 cell lines and partial downregulation in the CAL-51 knockdown line (Suppl. Fig. S3b). Expression of classic RAS proteins examined by Western blot with a pan-RAS antibody was not affected.

Deletion of the *RRAS2* gene decreased by 3-fold the proliferation rate of BT-549 cells in vitro and by 2-fold the proliferation of MCF7 cells, whereas *RRAS2* knockdown in CAL-51 cells reduced cell proliferation by 2-fold (Suppl. Fig. S3c). The three knockout or knockdown cell lines and their controls were orthotopically inoculated into the left inguinal mammary gland of immunodeficient *RAG2*^−/−^γc^−/−^ female mice. The effect of *RRAS2* depletion, or *RRAS2* reduction, in BT-549 and CAL-51 cells, respectively, was of an almost complete abolition of tumor growth in vivo (Suppl. Fig. S3d). The effect of R-RAS2 reduction on MCF7 growth in vivo was however less prominent, perhaps suggesting that *RRAS2* is more important for the growth of TNBC than for luminal ones. This idea would be in line with the fact that the BC developed by mice overexpressing wild type *RRAS2* are TNBC [35]. Altogether, these data show that mutated *RRAS2*, when bearing activating mutations, and wild type *RRAS2*, when overexpressed, are required for the growth in vitro and in vivo of human BC cells.

### R-RAS2 associates with multiple membrane receptors involved in breast cancer cell behavior

**Fig. 3:**
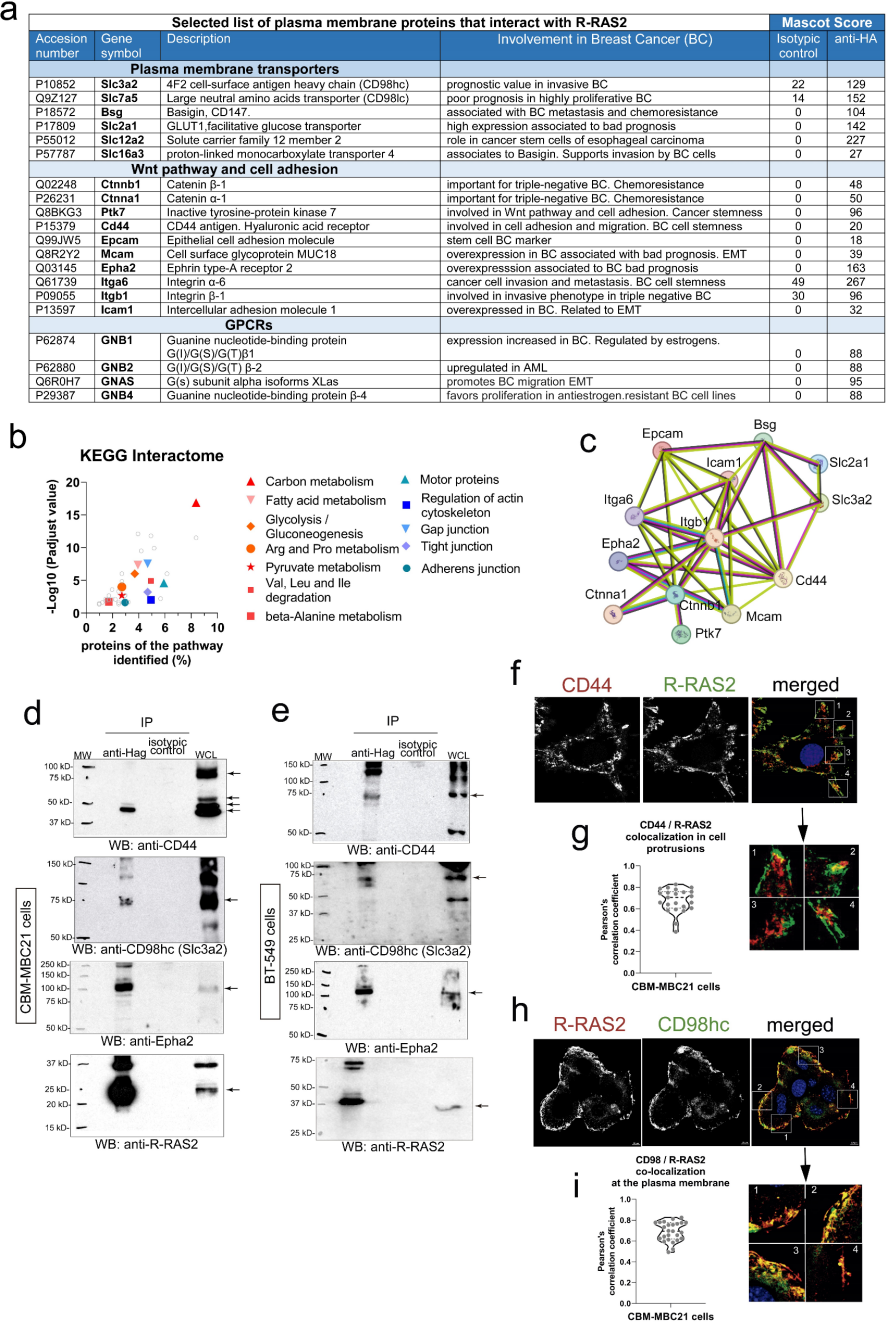
R-RAS2 interacts with plasma membrane receptors known to be important for breast cancer. **a**, Selected list of plasma membrane proteins that interact with R-RAS2 in a murine breast cancer tumor isolated from a Rosa26-*RRAS2*^fl/fl^ x Wap-Cre female mouse. Full data can be found in Extended Data Table 6. The known involvement of these proteins in cancer and breast cancer is briefly summarized, and the Mascot Score for the specific affinity column with anti-hemagglutinin (for hemagglutinin-tagged R-RAS2) and a control isotypic immunoglobulin column is also shown. Protein function and association with cancer data have been retrieved from The Human Protein Atlas: https://www.proteinatlas.org. **b**, Biological processes and pathways found to be significantly altered after KEGG analysis of the R-RAS2 plasma membrane interactome of Extended Data Table 6. The x-axis shows the percentage or proteins in the pathway found associated to R-RAS2 and the y-axis the–Log10 of the P adjusted value. Full data is provided in Extended Data Table 7. **c**, STRING network of physical and functional interactions among the indicated proteins from Fig. 3a. Pink lines indicate experimentally-determined interactions; blue lines indicated interactions found in curated databases; green lines indicate interactions found by textmining and black lines indicate co-expression. **d**, Co-immunoprecipitation of CD44, Epha2 and Slc3a2 (CD98hc) with R-RRAS2 (IP anti-Hag) was studied in detergent lysates of the CBM-MBC21 mouse cell line. The membranes were re-probed with anti-Hag as a control for loading of R-RAS2, and the arrows indicate the positions of the co-immunoprecipitated proteins and those in the whole cell lysate (WCL). The molecular weight markers are shown on the left. **e**, Co-immunoprecipitation of CD44, Epha2 and Slc3a2 (CD98hc) with R-RRAS2 in detergent lysates of the human breast cancer cell line BT-549 transfected with a Hag-tagged R-RAS2 construct. Legend as in panel *d*. **f**, Mid-plane confocal microscopy sections of CBM-MBC21 cells fixed and stained with R-RAS2 (anti-Hag) and anti-CD44 antibodies to show their co-localization at the plasma membrane. The nucleus of the cells is stained with DAPI (blue). Details of 4 areas of the plasma membranes are shown to the right to show the co-localization of R-RAS2 with CD44 in cell protrusions. **g**, Co-localization of R-RAS2 and CD44 was measured by analysis of all pixels in 23 cell protusions as in the inset of Fig. 3f and calculating the Pearson’s correlation coefficient. The violin plot shows all data points, the median (= 0.70) and the 75% and 25% percentiles. **h**, Mid-plane confocal microscopy sections of CBM-MBC21 cells fixed and stained with R-RAS2 (anti-Hag) and anti-CD98hc antibodies to show their co-localization at the plasma membrane. The nucleus of the cells is stained with DAPI (blue). Details of 4 areas of the plasma membranes are shown to the right to show the co-localization of R-RAS2 with CD98hc at the external (apical) plasma membrane of the cell aggregate. **i**, Co-localization of R-RAS2 and CD98hc was measured by analysis of all pixels in 25 membrane regions as in the inset of Fig. 3h and calculating the Pearson’s correlation coefficient. The violin plot shows all data points, the median (= 0.69) and the 75% and 25% percentiles

Once determined the *RRAS2*-dependence of human and mouse BC cells, we investigated the mechanisms by which R-RAS2 could be mediating cell transformation. We have described that R-RAS2 constitutively associates with antigen receptors, the BCR and TCR, in normal B and T cells, respectively [48]. More recently, we found that overexpressed wild type R-RAS2 associates with the BCR in leukemic CLL cells [36]. This led us to assess whether R-RAS2 could also bind to plasma membrane proteins in BC cells. To identify plasma membrane proteins that interact with R-RAS2 in BC, we disaggregated a freshly isolated BC tumor from a Rosa26-RRAS2^fl/fl^ x Wap-Cre mouse and surface biotinylated the cells with a cell-impermeable reagent. R-RAS2-interacting proteins associated with the plasma membrane were purified by two-step affinity chromatography and were identified by mass spectrometry (see Methods and Suppl. Fig. S4a). A total of 245 cell surface-associated proteins appeared to bind specifically to R-RAS2 (Extended Data Table 5), including plasma membrane proteins but also proteins of the actin and tubulin cytoskeletons and proteins involved in vesicular trafficking and organelle components, that could be indirectly associated to plasma membrane proteins. Of the plasma membrane proteins, some could be functionally classified as solute transporters, other as elements of the Wnt/β-catenin pathway, and other as cell-cell adhesion or extracellular matrix (ECM)-associated proteins. There was also a group of GPCRs (G-protein coupled receptors: Fig. 3a)-associated proteins. Interestingly, the Wnt-signaling pathway was already identified in the GOBP analysis of somatic mutations in transcribed genes (Fig. 1c) and in the IPA analysis of gene expression (Fig. 1e). Likewise, the fingerprint of interaction with the ECM was identified in the GOBP and KEGG analysis of gene mutations (Fig. 1c and d). A KEGG gene ontology analysis of the proteins associated with R-RAS2 at the plasma membrane (Extended Data Table 5) aligned also with signatures of cell shape, cell-cell interactions, cell motility and actin cytoskeleton in addition to signatures of metabolism (Fig. 3b).

Interestingly, some of the plasma membrane receptors identified in the R-RAS2 interactome (Fig. 3a) have been associated with BC and its prognosis. These include basigin (Bsg, CD147), β-catenin (Ctnnb1), and α-catenin (Ctnna1), which are linked to chemoresistance; β-catenin, Ptk7, CD44, and Epcam, which are associated with BC cell stemness; and CD98 (Slc3a2, 4F2), LAT1 (Slc7a5), GLUT1 (Slc2a1), and Epha2, which correlate with poorer prognosis [12]. We selected eight of these receptors to construct a STRING functional protein association network and found that all are interconnected within a first shell of interactors, either through experimental evidence or curated databases (Fig. 3c).

We next set out to validate some of the interactions detected in the interactomics screen by co-immunoprecipitation. Due to the relationship between R-RAS2 and degradation of valine, leucine and isoleucine detected after KEGG’s analysis of the interactome (Fig. 3b), we chose to investigate in this work the interaction with the large neutral amino acid transporter CD98lc/CD98hc (*Slc7a5/Slc3a2*). The light subunit CD98lc (*Slc7a5*, also known as LAT1) is overexpressed in BC and more significantly in TNBC and is an activator of cell metabolism and protein translation through the activation of the mTORC1 complex [18, 49]. In addition, we chose to investigate the interaction of R-RAS2 with CD44 because it is considered an important marker of cancer stemness, because it is involved in EMT and because it promotes metastasis through interaction with the ECM, most importantly with hyaluronic acid [50–52]. Another reason was that in our GOBP and KEGG analyses of somatic mutations in tumors derived from Rosa26-RRAS2^fl/fl^ x Wap-Cre mice, we found a fingerprint of alterations in the interaction with the ECM (Fig. 1c and d).

To validate by co-immunoprecipitation the interactions of R-RAS2 with CD44 and CD98hc/LAT1 found by proteomics, we first used the murine CBM-MBC21 cell line. Since this cell line derives from a tumor isolated in R26-RRAS2^fl/fl^ x Wap-Cre mice and in these mice the human R-RAS2 is tagged at the N-terminus with an hemagglutinin (Hag) epitope [36], associated proteins could be recovered by immunoprecipitation with anti-Hag and identified by probing western blots with different antibodies. We found that CD44 and CD98hc co-immunoprecipitated specifically with R-RAS2 (Fig. 3d), confirming the association of these membrane receptors with R-RAS2. In regard to the CD44/R-RAS2 interaction, several forms of different molecular weights were found in the whole cell lysate (Fig. 3d). CD44 is known to be expressed in multiple forms resulting from alternative splicing [51]. Of those, the standard splice form (CD44s) is reported to migrate with a molecular weight of 80–90 kDa [53]. A protein of that molecular weight is detected in the whole cell lysate (upper arrow, Fig. 3b) but not in the anti-Hag-R-RAS2 immunoprecipitate. To characterize the CD44 form that is expressed by CBM-MBC21 cells, we amplified and sequenced the mRNA transcripts corresponding to CD44 in the CBM-MBC21 cell line and found that these cells express an alternatively-spliced form lacking exons 6–15, all of which encode portions of the extracellular domain (Suppl. Fig. S4b), and that corresponds to CD44s. Therefore, the CD44 spliced form expressed in CBM-MBC21 is the standard splice form CD44s. We propose that the lower molecular weight of CD44s associated to R-RAS2 in the CBM-MBC21 cell line must be due to differences in posttranslational modifications, particularly glycosylation, since glycoproteomic studies have previously reported a low glycosylation form of CD44s associated to heightened hyaluronan binding capacity and promotion of invasive properties [54, 55]. Of note, CD44s may promote BC cell survival through the activation of the PI3K/Akt pathway, a well-known direct effector pathway of R-RAS2 [56–58].

To investigate if R-RAS2 also associated with the aforementioned membrane receptors in human BC cells, we used the TNBC BT-549 cell line after it was transiently transfected with Hag epitope-tagged R-RAS2. In western blots of the proteins pulled-down with the anti-Hag antibody, R-RAS2 was seen to associate with CD44 and CD98hc (Fig. 3e). These two membrane receptors co-purified with R-RAS2, indicating that R-RAS2 interacted with them either directly or through another linking protein. Interestingly, the CD44 associated with R-RAS2 in BT-549 cells had a molecular weight below 80 kDa, although larger than the CD44s form found in the murine cell line (Fig. 3e). We did not investigate further the exon composition of CD44 expressed in BT-549 cells.

Another important receptor in BC development is the receptor tyrosine kinase Epha2 which is frequently found overexpressed [59]. Epha2 was also found to interact with R-RAS2 in the interactomics data (Fig. 3a) and by co-immunoprecipitation in CBM-MBC21 (Fig. 3d) and in BT-549 cells (Fig. 3e). Interestingly, several membrane receptors found to associate directly or indirectly with R-RAS2 in our interactomics data (Fig. 3a) have been shown to interact among them (Fig. 3c). Thus, CD98hc does not only form a covalent dimer with CD98lc (LAT1, *Slc7a5*, Fig. 3a) it does also associate to CD147/basigin (*Bsg*, Fig. 3a) and β1-integrins (*itgb1*, Fig. 3a) [15, 16]. Moreover, CD147/basigin not only interacts with CD98hc, it has also been shown to form complexes with CD44 and with ErbB family members [60]. Thus, it could be hypothesized that rather than forming binary complexes with the membrane receptors of Fig. 3a, R-RAS2 could be forming part of multi-receptor signaling complexes at the plasma membrane.

We decided to further investigate the role of R-RAS2 on the function of the CD98hc/LAT1 transporter and on the function of CD44; leaving the study of the role of R-RAS2 downstream of Epha2 for a future study. To study the possible functional role of R-RAS2 downstream of CD44 and CD98hc/CD98lc, we first tried to validate the co-immunoprecipitation data (Fig. 3d and e) by confocal microscopy, i.e. analyzing if R-RAS2 co-localized with CD98hc and with CD44 when the structure of the cells is preserved. We previously described that R-RAS2 strongly concentrates in focal adhesions [61]. We now found that CD44 and R-RAS2 strongly co-localize at the plasma membrane and especially in cell protrusions in CBM-MBC21 cells, which could be relevant for a possible role for R-RAS2 in CD44-promoted cell migration. (Figure 3f and g). Likewise, R-RAS2 was shown to strongly co-localize with CD98hc in CBM-MBC21 cells at membrane ridges (Fig. 3h and i). These data reinforce the co-immunoprecipitation data suggesting that R-RAS2 is associated to CD44 and CD98hc in the plasma membrane of BC cells.

### R-RAS2 is necessary for the activation of breast cancer cell metabolism and the mTOR pathway via the large neutral amino acid transporter

**Fig. 4:**
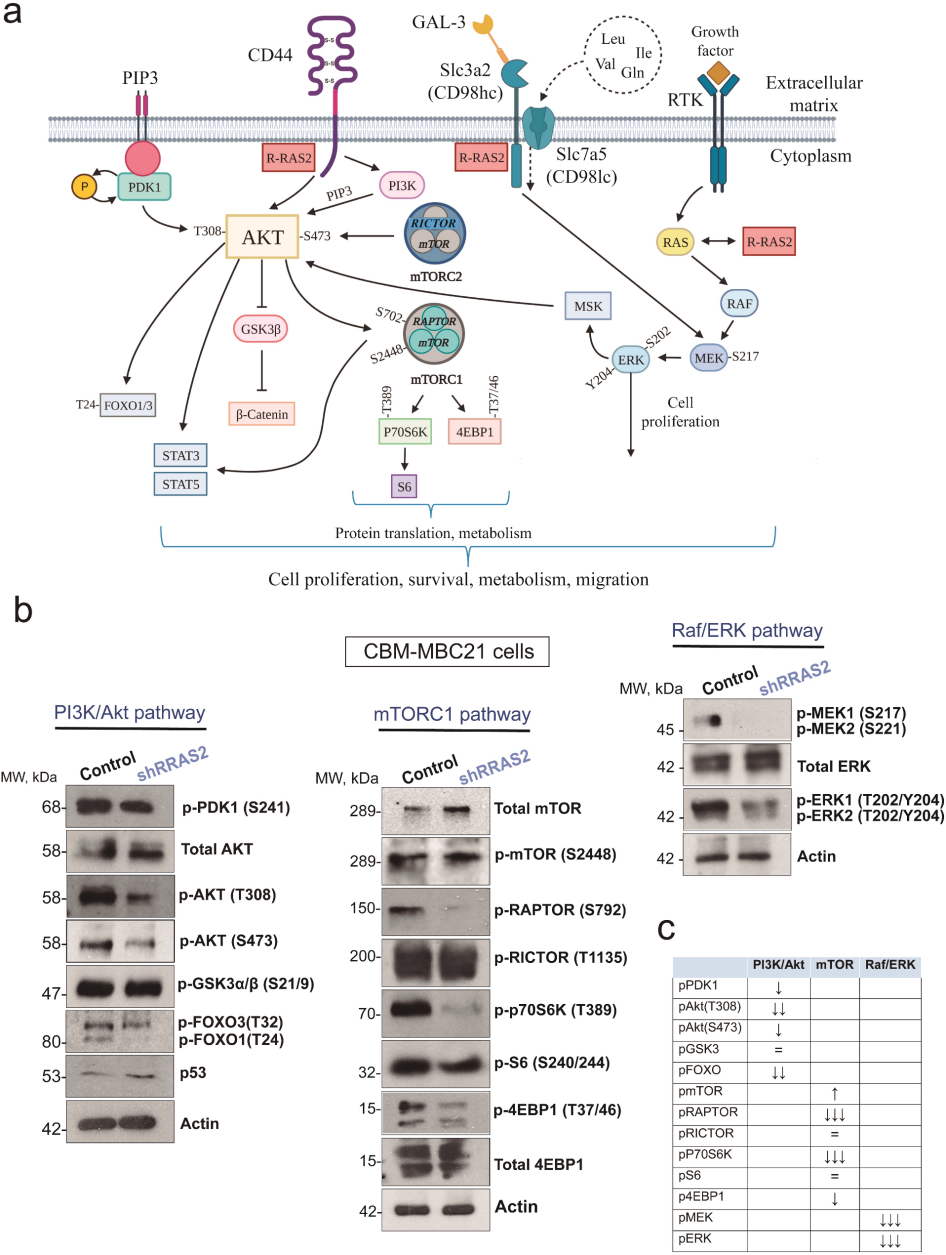
R-RAS2 controls PI3K/Akt, ERK/MAPK and mTORC1 pathway activation in murine RRAS2-overexpressing breast cancer cells. **a**, Cartoon illustrating RAS/ERK/MAPK, PI3K/Akt and mTORC1 pathway activation by Receptor Tyrosine Kinases (RTKs) like EphaA2, EGFR or HER2, and by amino acid transporters like the CD98hc/CD98lc(LAT1) complex, and by CD44. The positions of some relevant phosphorylated residues are indicated, as well as the putative position of R-RAS2 downstream of the RTKs, CD44 and CD98. **b**, Western blot analysis of signaling pathway activity based on the phosphorylation of key residues in the elements indicated. Post-nuclear cell lysates of wild type CBM-MBC21 cells and a shRNA-generated *RRAS2* knockdown of that cell line were analyzed in the blots. **c**, Summary of the results generated by Western blot. The inhibitory effect of R-RAS2 depletion is indicated by the number of arrows pointing downwards

We have previously shown using human MDA-MB-231 breast cancer cell line that *RRAS2* knockdown reduces phosphorylation of proteins in the PI3K/Akt/mTORC1 pathway and that R-RAS2 is necessary to maintain BC cell metabolism and protein translation [47]. Since R-RAS2 interacts in the murine CBM-MBC21 cell line with the neutral amino acid transporter CD98hc/CD98lc, which is involved in the activation of the mTOR pathway and cell metabolism, with CD44, which is known to activate Akt, and with receptor tyrosine kinases (RTKs) such as Epha2, which activate the Raf-ERK MAPC pathway (Fig. 3), we tentatively place R-RAS2 as intracellularly associated with CD44, the amino acid carrier CD98hc/CD98lc and RTKs (Fig. 4a). In this cartoon we also indicate some of the phosphorylation sites in key proteins of the Raf/ERK, PI3K/Akt, and mTOR pathways that could be altered in the absence of R-RAS2 downstream of the mentioned membrane receptors.

By comparing the parental CBM-MBC21 cell line and its *RRAS2* knockdown derivative by Western blot analysis, we aimed to investigate if high R-RAS2 expression is needed in the murine BC cell line to sustain the activity of the three pathways when cells are cultured in full standard medium. We found that R-RAS2 depletion reduced the phosphorylation of effector proteins at sites compatible with its participation in the PI3K/Akt, mTOR and Raf/ERK pathways (Fig. 4b, summary in Fig. 4c). With some differences in specific phosphorylation sites, the effect of R-RAS2 depletion in the human BT-549 breast cancer cell line also involved depressing the activity of the Raf/ERK, PI3K/Akt, and mTOR pathways (Suppl. Fig. S5a and S5b).

**Fig. 5:**
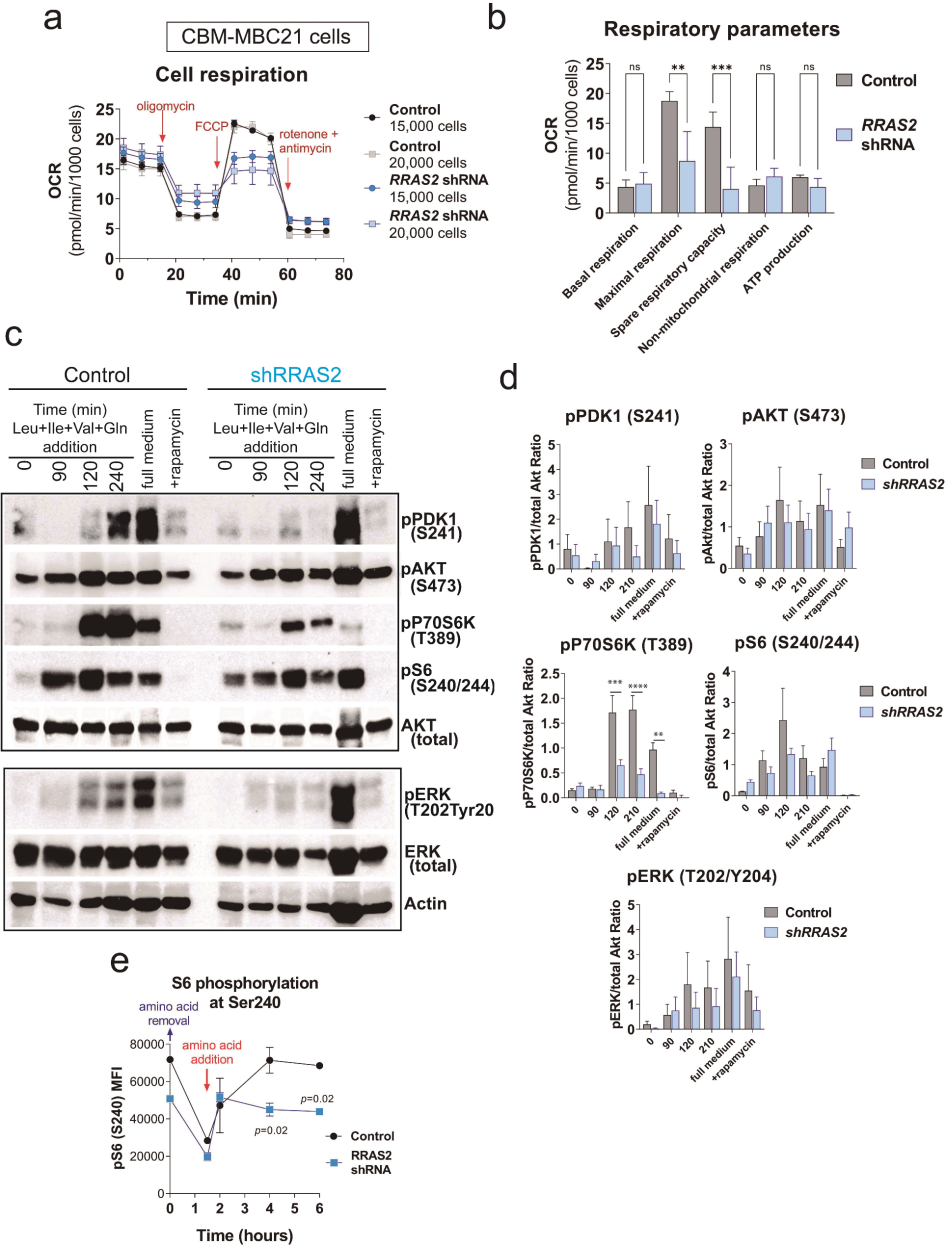
R-RAS2 is functionally downstream of CD98/LAT1 controlling mTORC1 activation and breast cancer cell metabolism. **a**, Seahorse analysis of the oxygen consumption rate (OCR) of WT and *RRAS2* knockdown CBM-MBC21 cells in complete medium. Measurements were made in sextuplicate after plating the two types of cell at different densities (15,000 cells/well and 20,000 cells/well). The drugs (oligomycin, FCCP and rotenone + antimycin A) were added sequentially to measure different respiratory parameters. Datapoints show the mean ± s.e.m. **b**, Respiratory parameters calculated from the Seahorse data (Fig. 5a), the significance of which was assessed with a two-way ANOVA and Sidak’s multiple comparison test. **, *p* = 0.004; ***, *p* = 0.0009. **c**, Western blot analysis was performed to assess signaling pathway activity by examining the phosphorylation of key residues in the indicated elements. Control and CBM-MBC21 knockdown cells were deprived of the amino acids Leu, Ile, Val, and Gln for 1.5 h, followed by replenishment of the specified amino acids for the indicated time points (in minutes). Post-nuclear cell lysates were analyzed via Western blotting. As controls, lysates from cells maintained in full medium throughout the experiment and from cells treated with 50 nM rapamycin in full medium were included. **d**, Quantification by densitometry of Western blots as in Fig. 5c run in triplicate. Bar plots show the mean ± s.e.m of densitometry data referring the intensity of the phosphoprotein bands to that of total Akt. Significance was assessed using a two-way ANOVA test. **, *p* = 0.002; ***, *p* = 0.0002; ****, *p* < 0.0001. **e**, Effect of amino acids Leu, Ile, Val and Gln deprivation followed by replenishment on the phosphorylation of S6 protein at Ser240 analyzed by flow cytometry. Datapoints represent the mean ± s.e.m of triplicates. Significance was assessed using a two-way ANOVA Sidak’s multiple comparison test

In *Rras2*-null mice we have shown that R-RAS2 controls B cell’s metabolism by regulating the PI3K/Akt/mTORC1 pathway and the replication, transcription and function of mitochondria [56]. Therefore, we aimed to investigate if the reduced activity of the mTORC1 pathway in the R-RAS2 knockdown CBM-MBC21 breast cancer cells caused an alteration of mitochondrial activity. Thus, we first investigated whether R-RAS2 depletion changed the capacity of mitochondria to consume oxygen. By measuring oxygen variations with an oxygen probe in response to the serial addition of different inhibitors, we determined that R-RAS2 reduction in CBM-MBC21 cells did not affect basal respiration but strongly inhibited the maximal respiration rate and the spare respiratory capacity (Fig. 5a and b). These data suggest that R-RAS2 reduction makes CBM-MBC21 cells unable to cope with situations that require increased mitochondrial respiration and energy demands.

We also analyzed the effect of R-RAS2 depletion on the use of alternate substrates for ATP production using a Mitofuel test. The knockdown cells were more sensitive to the depletion of glucose than control ones since inhibition of glucose oxidation with UK5099 was not compensated in the presence of both glutamine and fatty acids (Suppl. Fig. S6a). Inhibition in the use of glutamine or fatty acids was compensated if glucose could be utilized. These results suggest that high R-RAS2 expression makes CBM-MBC21 cells highly dependent on glucose for mitochondrial respiration and less dependent on glutamine and fatty acid consumption. Thus, although the KEGG analysis of the R-RAS2 plasma membrane interactome of a freshly-isolated tumor identified a fingerprint of fatty acid metabolism (Fig. 3b), the CBM-MBC21 cell line seems to be independent of fatty acid metabolism, at least in culture.

Since the amino acid carrier CD98hc/CD98lc is an activator of mTORC1 independently of Akt activity and under the control of different small GTPases [62], we next proceeded to determine what was the effect of R-RAS2 depletion on the activation of mTORC1 promoted by the transport of large neutral amino acids. First, we established that knocking down *RRAS2* did not alter the expression of CD98hc at the plasma membrane of CBM-MBC21 cells (Suppl. Fig. S6b). Subsequently, we deprived WT and *RRAS2* knockdown CBM-MBC21 cells of the amino acids Leu, Ile, Val, and Gln, and analyzed the effect of adding back those amino acids on PI3K and mTORC1 downstream effectors by Western blot. Large neutral amino acid deprivation strongly reduced the phosphorylation of p70S6K and its substrate S6 in WT and knockdown cells (Fig. 5c and d). However, whereas the replenishment of those amino acids elicited an upregulation of the phosphorylation of these two mTORC1 effectors in WT cells, such upregulation was markedly less intense in *RRAS2* knockdown cells (Fig. 5c and d). A similar effect was visualized in the human BT-549 *RRAS2* KO cells compared to WT controls. In BT-549 cells, the effect of *RRAS2* KO on the phosphorylation of the PI3K effector PDK1 and the mTORC2 phosphorylation of Akt in response to large amino acid replenishment was clearly reduced compared to WT cells (Suppl. Fig. S7a and S7b). In contrast to BT-549, R-RAS2 deprivation in CBM-MBC21 cells caused a defective upregulation of PDK1 phosphorylation but not of Akt phosphorylation upon adding back the missing amino acids (Fig. 5c). Taking advantage on the suitability of the anti-phospho-S6 (Ser240/244) antibody for phosFlow analysis, we repeated the amino acid deprivation and replenishment experiment to be analyzed by this method. We found that R-RAS2-deficient CBM-MBC21 cells were quite unresponsive to the increase in S6 phosphorylation induced by the transport of these amino acids by CD98hc/CD98lc (Fig. 5e). Likewise, RRAS2 knockout BT-549 cells were deficient in the upregulation of S6 phosphorylation detected by phosFlow cytometry upon addition of large neutral amino acids (Suppl. Fig. S7c). These results further strengthened the idea that R-RAS2 mediates signaling via the CD98hc/CD98lc (LAT1) large neutral amino acid transporter, leading to mTORC1 activation.

### R-RAS2 is required for the rearrangement of the actin cytoskeleton promoted by CD44 and the formation of membrane ridges

CD44 is the main receptor for hyaluronic acid (HA) which is on the other hand the major extracellular matrix component. It is involved in maintaining cancer cell stemness and in promoting EMT and adoption of a metastatic behavior by cancer cells [20, 52]. Interestingly, the CD44 standard splice form (CD44s), but not variant forms, positively associates with BC cell stemness [63]. The murine CBM-MBC21 cells express the CD44s form (Fig. 3d and Suppl. Fig. S4b). To investigate if R-RAS2 is downstream of CD44 mediating BC cell migration and metastasis, we first determined that R-RAS2 knockdown in CBM-MBC21 cells did not alter the expression of CD44, suggesting that all subsequent effects of reducing R-RAS2 expression were not due to reduction of the expression of the receptor (Suppl. Fig. S8a).

**Fig. 6:**
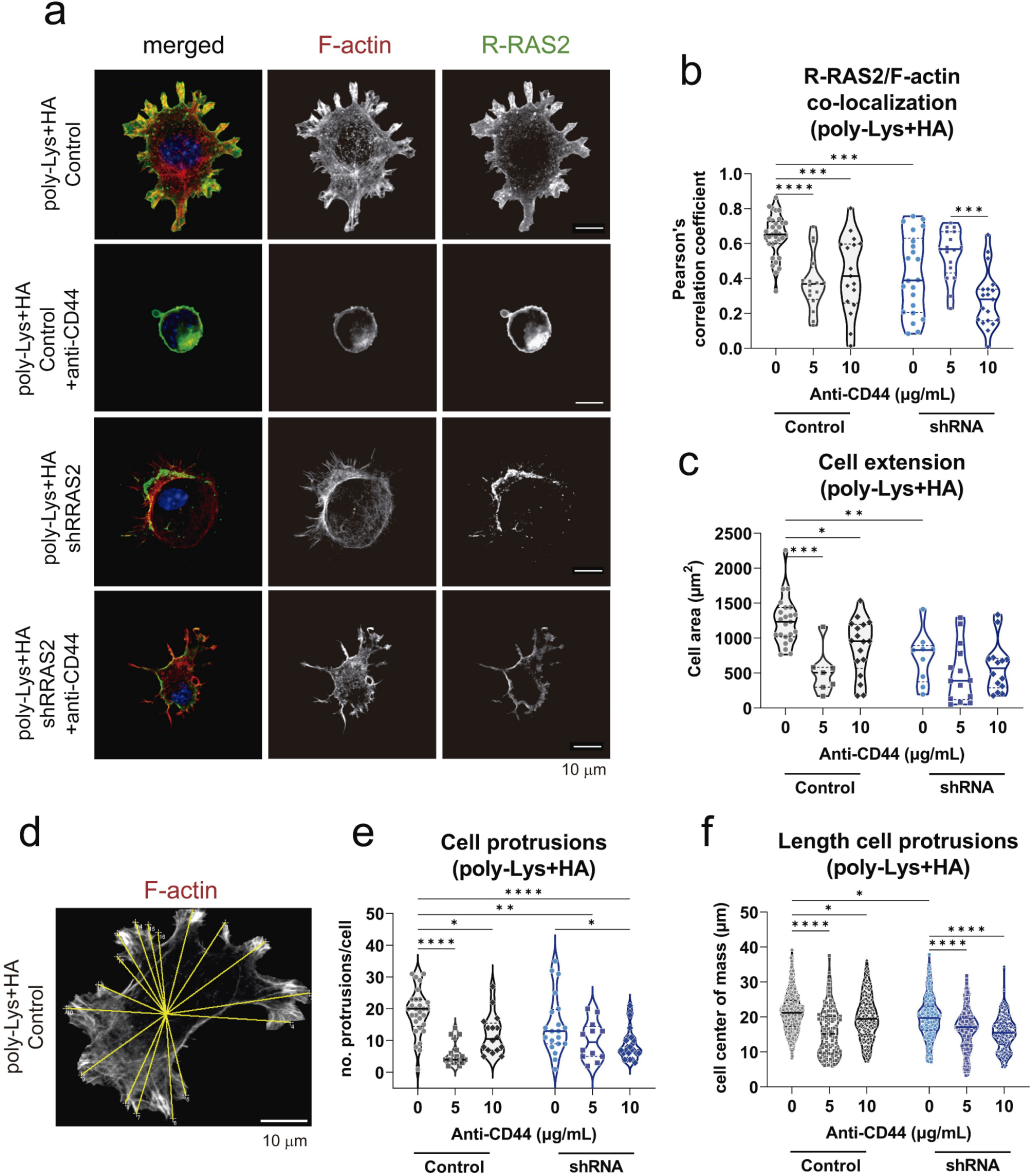
R-RAS2 co-localizes with CD44 in F-actin-rich cell protrusions of breast cancer cells. **a**, Confocal microscopy sections at the plane of contact with the coverslips of CBM-MBC21 control and knockdown cells plated on coverslips coated with poly-L-lysine alone or with poly-L-lysine plus hyaluronic acid (HA). Cells were plated in the presence or absence of a blocking anti-CD44 antibody. After incubation, cells were fixed and stained with R-RAS2 (anti-Hag) and phalloidin to show their co-localization at sites rich in F-actin. The nucleus of the cells is stained with DAPI (blue). **b**, Co-localization of R-RAS2 and F-actin at cell protrusions was measured by analysis of all pixels in 17–33 cell protrusions and calculating the Pearson’s correlation coefficient. The violin plot shows all data points, the median and the 75% and 25% percentiles for each condition. Statistical significance was assessed using a one-way ANOVA Tukey’s multiple comparison test. ***, *p* < 0.001, ****, *p* < 0.0001. **c**, Cell area at the contact site with the coverslip in the experiment of Fig. 6a-b, was calculated for 7–28 cells per condition. The violin plot shows all data points, the median and the 75% and 25% percentiles for each condition. Statistical significance was assessed using a one-way ANOVA Tukey’s multiple comparison test. *, *p* = 0.03; **, *p* = 0.008; ***, *p* = 0.0003; ****, *p* < 0.0001. **d**, The number and distance to the center of cell protrusions were measured in phalloidin stains by calculating the center of mass and drawing lines (yellow) from the center to the tip of cell protrusions. **e**, The number of protrusions was calculated as in Fig. 6d for a minimum of 15 cells per culture condition. The violin plot shows all data points, the median and the 75% and 25% percentiles for each condition. Statistical significance was assessed using a one-way ANOVA Tukey’s multiple comparison test. *, *p* < 0.05; **, *p* = 0.002; ****, *p* < 0.0001. **f**, The distance to the cell center of mass of protrusions was calculated as in Fig. 6d for a minimum of 100 cell protrusions per culture condition. The violin plot shows all data points, the median and the 75% and 25% percentiles for each condition. Statistical significance was assessed using a one-way ANOVA Tukey’s multiple comparison test. *, *p* < 0.05; ****, *p* < 0.0001

To study the possible functional role of R-RAS2 downstream of CD44, we engaged in a series of confocal microscopy experiments after plating CBM-MBC21 cells on coverslips coated with different polymers. The finding that CD44 and R-RAS2 strongly co-localize at the plasma membrane in cell protrusions of CBM-MBC21 cells (Fig. 3f and g) could be relevant for a possible role for R-RAS2 in CD44 promoted cell migration. To determine if the formation of such membrane protusions requires CD44 and R-RAS2, we carried out confocal microscopy analysis of CBM-MBC21 cells seeded on coverlips coated with poly-L-lysine and hyaluronic acid. Control cells expressing full dose of R-RAS2 formed F-actin-decorated membrane protrusions (Fig. 6a) in which F-actin and R-RAS2 strongly co-localized (Fig. 6b). The cells spread on the HA-decorated surface and such spreading was reduced if *RRAS2* was knocked down or if cells were incubated with the blocking anti-CD44 antibody (Fig. 6a and c). These results suggest that both CD44 and R-RAS2 mediate the spreading of CBM-MBC21 cells on HA-coated surfaces.

In addition to calculating cell extension in the different culture conditions, we used a custom Fiji macro to calculate the mass center of each cell and from there the number of cell protrusions and their distance to the cell center (Fig. 6d). The use of the blocking anti-CD44 antibody caused a reduction of the number (Fig. 6e) and length (Fig. 6f) of cell protrusions. Likewise, *RRAS2*-knockdown CBM-MBC21 cells were defective in the formation of lengthy cell protrusions (Fig. 6a, e and f). Altogether, these results suggest that R-RAS2 is required for the CD44-promoted formation of cell protrusions that involve the rearrangement of the actin cytoskeleton.

### R-RAS2 is activated at membrane ridges and mediates PI3K/Akt activation downstream of CD44

**Fig. 7:**
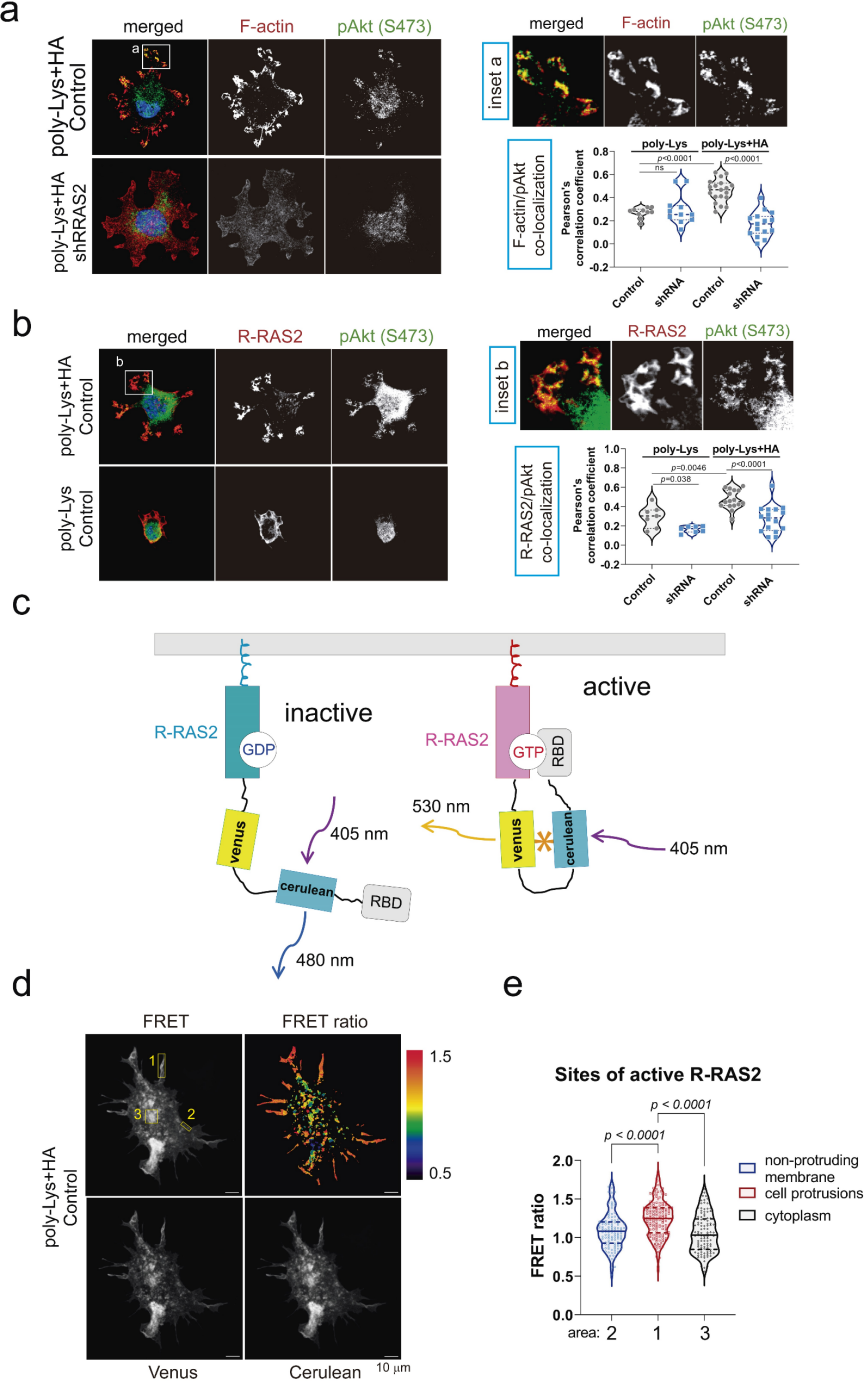
Cell protrusions are sites of high R-RAS2 activity and Akt phosphorylation. **a**, Confocal microscopy sections at the plane of contact with the coverslips of CBM-MBC21 control and knockdown cells plated on coverlips coated with poly-L-lysine plus hyaluronic acid (HA). After incubation, cells were fixed and stained with anti-phospho-Akt (S473) and phalloidin to show their co-localization at sites rich in F-actin. The nucleus of the cells is stained with DAPI (blue). A detail of inset *a* is shown to the right to illustrate strong co-localization at cell protrusions. Co-localization of F-actin and phospho-Akt was measured by analysis of all pixels in 10–19 cell protrusions per condition and calculating the Pearson’s correlation coefficient. The violin plot shows all data points, the median and the 75% and 25% percentiles. Statistical significance was assessed by carrying out Mann-Whitney tests. **b**, Confocal microscopy sections at the plane of contact with the coverslips of CBM-MBC21 control and knockdown cells plated on coverlips coated with poly-lysine plus hyaluronic acid (HA) or poly-L-lysine alone. After incubation, cells were fixed and stained with anti-phospho-Akt (S473) and with anti-Hag (for R-RRAS2) to show their co-localization at cell protrusions. The nucleus of the cells is stained with DAPI (blue). A detail of inset *b* is shown to the right to illustrate strong co-localization at cell protrusions. Co-localization of R-RAS2 and phospho-Akt was measured by analysis of all pixels in 7–16 cell protrusions per condition and calculating the Pearson’s correlation coefficient. The violin plot shows all data points, the median and the 75% and 25% percentiles. Statistical significance was assessed by carrying out Mann-Whitney tests. **c**, Schematic of the R-RAS2 biosensor designed to measure its active form (GTP-bound) in living cells. Human R-RAS2 was appended a FRET donor (cerulean) and a FRET acceptor (venus) to its N-terminal end. Cerulean is preceded at its N-terminus by the Ras-binding domain (RBD) of the catalytic subunit of PI3Kδ (p110δ). The exchange of GDP by GTP in R-RAS2 allows the RBD to bind the effector loops of R-RAS2 and brings cerulean into closer proximity to venus, allowing an increase in FRET efficiency. **d**, Confocal microscopy sections at the plane of contact with the coverslips of CBM-MBC21 control cells plated transfected with the R-RAS2 biosensor and plated on coverslips coated with poly-L-lysine plus hyaluronic acid (HA). The example shows one cell with protruding cell extensions and the fluorescence of the biosensor in the cerulean, venus and FRET channels. The colored micrograph shows the FRET ratio resulting of dividing the fluorescence intensity in the FRET channel by this of the cerulean channel. The colored bar to the right shows the scale of FRET ratios. The FRET ratio was calculated for three types of cell sites: plasma membrane at cell protrusions (1), non-protruding plasma membrane (2) and cytoplasm (3). **e**, Violin plot showing all data points, the median and the 75% and 25% percentiles of the FRET ratios calculated in 109–150 cell sites of the three types in a total of seven cells. Statistical significance was assessed using a one-way ANOVA Tukey’s multiple comparison test

The cell protrusions formed by CBM-MBC21 cells that are rich in F-actin do also present an accumulation of phospho-Akt (Ser473) that is dependent on the presence of HA (Fig. 7a). Those protrusions did also present an enrichment in R-RAS2 that co-localized with pAkt (Fig. 7b). Since R-RAS2 is upstream of the PI3K/Akt pathway in CBM-MBC21 cells (Fig. 4) and interacts with CD44 (Figs. 3 and 6), we hypothesized that R-RAS2 becomes activated at membrane ridges and promotes the activation of the PI3K/Akt pathway and perhaps the polymerization of actin.

To determine if R-RAS2 not only accumulates at membrane ridges in CBM-MBC21 cells but also it is in the active form in such locations, we transfected those breast cancer cells with a recently published FRET biosensor used to determine the activity of R-RAS2 in T cell and B cell lymphomas [64]. The biosensor contains a FRET donor and a FRET acceptor in the same molecule appended to the N-terminus of R-RAS2 together with the RAS-binding domain (RBD) of the p110δ subunit of PI3Kδ (Fig. 7c). In the Active, GTP-bound stated of R-RAS2, the RBD is able to bind R-RAS2, this bringing the FRET donor Cerulean closer to the FRET acceptor Venus. This results in increased sensitized emission by Venus when Cerulean is excited at 405 nm. Using CBM-MBC21 cells transfected with the biosensor and plated on a poly-Lys + HA-coated surface, we measured the sensitized emission of Venus when Cerulean was excited (FRET intensity) as well as the direct emission by Cerulean and direct emission of Venus (when Venus was directly excited) in different regions of interest (ROIs) in the cells. In addition, a FRET ratio was calculated that resulted from the division, pixel-by-pixel, of the FRET intensity by the intensity of the Cerulean donor. This ratio corrected for the abundance of the protein and gave a better idea about the sites in the cell where R-RAS2 is more active (Fig. 7d). The calculation of the FRET ratio showed that R-RAS2 is more active at the protruding cell extensions than in other parts of the plasma membrane and than in internal locations (Fig. 7d and e).

### R-RAS2 mediates breast cancer migration and metastasis in a CD44-promoted manner

**Fig. 8:**
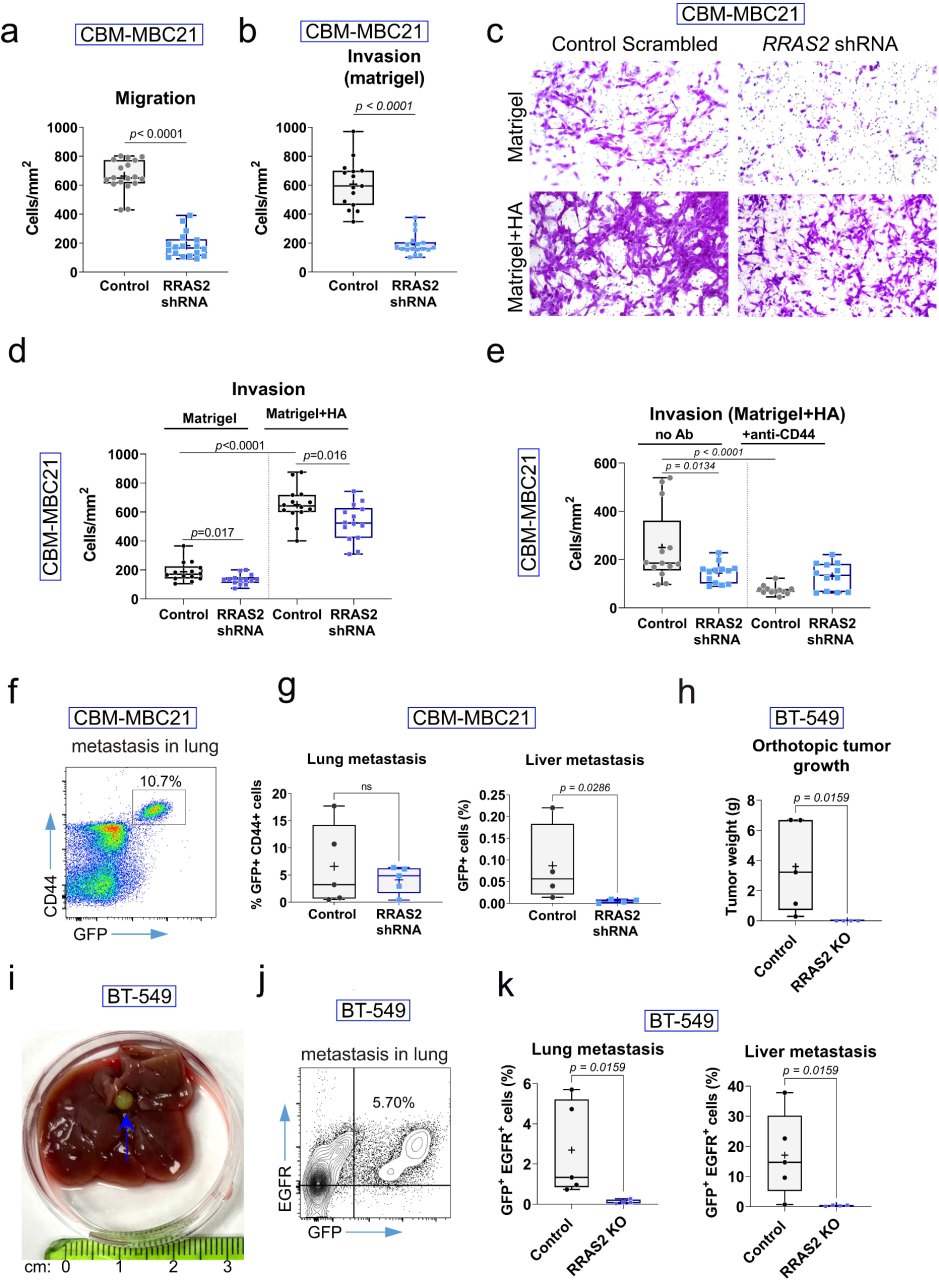
R-RAS2 is downstream of CD44 promoting migratory, invasive and metastatic behavior of breast cancer cells. **a**, Box and whiskers plot showing all experimental points of a migration assay in Boyden chambers separated by a 8 μm-diameter pore membrane. Cells were incubated overnight (~16 h). Each point represents the mean of cells per surface unit (mm^2^) of membrane counted. A number of 18 areas of the membrane were counted per experimental condition. Statistical significance was assessed by carrying out a Mann-Whitney test. **b**, Box and whiskers plot showing all experimental points of a migration assay in Boyden chambers separated by a 8 μm-diameter pore membrane and layered on the upper chamber with 100 μL of Matrigel. Cells were incubated overnight (~16 h). Each point represents the mean of cells per surface unit (mm^2^) of membrane counted. A number of 18 areas of the membrane were counted per experimental condition. Statistical significance was assessed by carrying out a Mann-Whitney test. **c**, Light field microscopy of the bottom part of the membrane with 8-μm diameter pores separating an upper Boyden chamber in which CBM-MBC21 control of knockdown cells were seeded in serum-free medium and a lower chamber containing medium with 20% feta bovine serum. In addition, the upper chamber had a layer or either Matrigel or Matrigel + hyaluronic acid directly on top of the membrane. Cells were allowed to migrate for 8 h from the top to the bottom chambers and through the Matrigel or Matrigel + HA layers. The bottom part of the membrane was stained with crystal violet to count the number of migrated cells. **d**, Box and whiskers plot showing all experimental points of the experiment illustrated in Fig. 8c. Each point represents the mean of cells per surface unit (mm^2^) of membrane counted. A number of 14 areas of the membrane were counted per experimental condition. Statistical significance was assessed using unpaired t-tests. **e**, An invasion assay as this of Fig. 8 b and d, was carried out in Boyden chambers coated in the upper chamber with a layer of Matrigel + hyaluronic acid. Control and knockdown CBM-MBC21 cells were incubated or not (no Ab) with 5 μg/mL of blocking anti-CD44 antibody in the upper chamber for 8 h. Box and whiskers plot showing all experimental points that represent the mean of cells per surface unit (mm^2^) of membrane counted. A number of 13–14 areas of the membrane were counted per experimental condition. Statistical significance was assessed using a one-way ANOVA Tukey’s multiple comparison test. **f**, The metastatic capacity of control and knockdown CBM-MBC21 cells 25 days after orthotopic inoculation in the left inguinal mammary gland of female C57BL/6 mice was assessed by flow cytometry of the lungs and liver. The pseudocolor plot shows the identification of metastatic breast cancer cells in the lungs according to the expression of GFP and CD44. **g**, Box and whiskers plot showing all experimental points calculated as illustrated in Fig. 8f and Suppl Fig. S7f referring to BC cell infiltration of lungs and liver, respectively. Each point represents a single mouse. Statistical significance was assessed by carrying out Mann-Whitney tests. **h**, Box and whiskers plot showing all experimental points indicating primary tumor weight in mice inoculated with BT-549 human BC cells at the day of sacrifice (day 39). Statistical significance was assessed by carrying out a Mann-Whitney test. **i**, Photograph of the entire liver taken from a mouse inoculated with wild type BT-549, control, BC cells. The blue arrow points at the presence of a green fluorescent BC tumor nodule. **j**, The metastatic capacity of control and knockdown BT-549 cells 39 days after orthotopic inoculation in the left inguinal mammary gland of female Rag2^−/−^γc^−/−^ mice was assessed by flow cytometry of the lungs and liver. The two-color plot shows the identification of metastatic breast cancer cells in the lungs according to the expression of GFP and human EGFR. **k**, Box and whiskers plot showing all experimental points calculated as illustrated in Fig. 8j referring to BC cell infiltration of lungs and liver. One out of four liver lobules per animal were processed for flow cytometry analysis. Each point represents a single mouse. Statistical significance was assessed by carrying out Mann-Whitney tests

To determine if R-RAS2 is functionally downstream of CD44 promoting BC cell migration, we engaged in a series of transwell assays in Boyden chambers in which CBM-MBC21 cells migrated from serum-free medium in the upper chamber to the serum-containing medium in the lower chamber passing through pores of 8 μm-diameter and through matrices of different composition. R-RAS2 knockdown CBM-MBC21 cells were first found to have a reduced rate of migration through the 8 μm-diameter pores compared to their control counterparts (Fig. 8a). The knockdown cells also showed impaired invasive capacity in an assay in which they needed to migrate through a layer of Matrigel in addition to the 8 μm-diameter pores before reaching the lower chamber (Fig. 8b). Matrigel is a solubilized membrane matrix, secreted by murine EHS sarcoma cells, rich in ECM components such as laminin, collagen IV and entactin [65]. Full depletion of *RRAS2* expression in the human BT-549 BC cells also resulted in a reduction of both migration through 8 μm-diameter pores and invasion through Matrigel (Suppl. Fig. S8b and S8c).

To determine if R-RAS2 depletion affected the invasiveness of CBM-MBC21 cells promoted by CD44, we repeated the transwell experiments using a mixture of Matrigel with CD44’s ligand HA. The presence of HA promoted the invasive capacity of the cells and, as in the absence of HA, invasion was decreased if *RRAS2* was knocked down (Fig. 8c and d). Migration through the Matrigel + HA matrix by CBM-MBC21 was inhibited by incubation of the cells with a blocking anti-CD44 antibody (Fig. 8e). This data suggested that R-RAS2 is required to promote CD44-mediated migration of BC CBM-MBC21 cells through extracellular matrices rich in hyaluronic acid and therefore, that R-RAS2 mediates intracellular signaling by CD44 during BC cell migration.

To explore if R-RAS2 promoted breast cancer cell metastasis in vivo, we carried out experiments of orthotopic inoculation of parental and *RRAS2*-knockdown CBM-MBC21 breast cancer cells into syngeneic C57BL/6 female mice. Sixteen days after inoculation, we noticed that unlike mice inoculated with the knockdown cells, mice inoculated with the parental cells were not gaining weight but losing it (Suppl. Fig. S8d). Mice were sacrificed and samples of their lungs and livers were sectioned and stained with hematoxylin/eosin in search of metastasis. We found a clear nodule of infiltrating tumoral cells within the parenchyma of the lung of one mouse inoculated with scrambled control CBM-MBC21 cells but none in mice inoculated with the *RRAS2*-knockdown cells (Suppl. Fig. S8e). The knock-in cassette to generate the R26-RRAS2fl/fl x Wap-Cre mice also expresses green fluorescent protein (GFP) thanks to a bicistronic construct strategy [36]. Taking advantage of this fact, we explored by flow cytometry if tumoral CBM-MBC21 cells had metastasized to the liver and lungs forming micrometastasis that were not detected by histochemistry. Using two-color flow cytometry we could detect CD44 + GFP + CBM-MBC21 cells in the lungs of mice inoculated with control cells (Fig. 8 f and g) and less in mice inoculated with knockdown cells, although differences were not significant (Fig. 8g). To detect metastasis in the liver, we could not use the CD44 marker because the background staining was too high, and we relied on the use of GFP (Suppl. Fig. S8f). Using GFP, we could determine that *RRAS2* knockdown CBM-MBC21 cells metastized significantly less to the liver than control cells expressing the full dose of *RRAS2* (Fig. 8g).

To reinforce the results generated with the murine CBM-MBC21 cell line using a human BC cell line, we inoculated orthotopically in the left inguinal mammary gland of RAG2^−/−^γc^−/−^ female mice either control or *RRAS2* knockout BT-549 cells. After 39 days, in the control group there were visible breast tumors that were close to reach the maximum ethically allowed size. After euthanasia, the primary tumors were weighed and found to be significantly bigger in the control group than in the group inoculated with knockout cells (Fig. 8h and Suppl. Fig. S8g). Indeed, in the latter group mammary glands had a normal appearance (Suppl. Fig. S8g). Before inoculation, BT-549 cells had been stably transfected with a vector expressing GFP. This made it easier to identify metastatic nodules in distal organs (Fig. 8i, blue arrow). At a glance, six metastatic nodules in liver, three in the ovaries and one in a distal breast (third fat pad, right side), were detected in mice inoculated with control cells and none in mice inoculated with the knockdown ones (Suppl. Fig. S8h). We next used flow cytometry to estimate the number of metastatic cells in different organs. To this end, we used GFP as a marker of BT-549 cells together with expression of the human EGFR (Fig. 8j). Using those markers, we found a significant difference in the degree of metastatic breast cancer BT-549 cell infiltration of the lungs and liver (Fig. 8k).

These results show that R-RAS2 is required for the formation of metastasis from an orthotopic location by murine breast cancer cells that initially originated as a consequence of *RRAS2* overexpression (Fig. 2), as well as by a human TNBC cell line that overexpresses wild type *RRAS2*.

## Discussion

In our previous study, we found that overexpression of unmutated *RRAS2* in mammary gland epithelial cells induces the development of ductal breast carcinomas of the TNBC type in a pregnancy-dependent manner [35]. In parallel with this cause-effect finding in the mouse model, we observed that wild-type *RRAS2* is overexpressed in more than 50% of breast cancer samples, including luminal A, luminal B, HER2-enriched, and TNBC subtypes, with the highest frequency (75%) and expression levels found in TNBC. These data suggest that wild-type *RRAS2* may contribute to the development of most breast cancers, including hormone receptor-positive ones. However, the fact that genetically modified mice overexpressing *RRAS2* do develop TNBC strongly supports a cause-effect relationship between *RRAS2* overexpression and TNBC, particularly those associated with pregnancy or the postpartum period. A prevailing hypothesis is that *RRAS2* overexpression may play a driver role in pregnancy-associated TNBC, while serving a supportive or secondary role in luminal breast cancers. Supporting this idea, our findings using three human BC cell lines—two of the TNBC type and one luminal A—indicate that *RRAS2* depletion has a greater impact on the growth of TNBC cells in orthotopic locations compared to luminal A cells.

In our previous work, we described the generation of breast cancer through overexpression of wild-type *RRAS2*[35]. In the present study, we aimed to investigate the mechanisms underlying this transformation and to further understand the role of R-RAS2 in breast cancer cell behavior. Although we previously used knockdown approaches to examine the effects of *RRAS2* depletion on the growth of human and murine BC cell lines both in vitro and in vivo [47], the derivation of the CBM-MBC21 cell line from a breast tumor that arose in a mouse engineered to overexpress *RRAS2* provided a unique opportunity to assess its relevance. This is because the original tumor directly resulted from *RRAS2* overexpression; an advantage not available in established BC cell lines, even if they overexpress *RRAS2*. We demonstrate here that reducing *RRAS2* expression four-fold in the CBM-MBC21 cell line slows proliferation in vitro due to a delayed progression through the S-phase of the cell cycle and diminishes the cells’ ability to form tumors in orthotopic locations. Additionally, we show that high *RRAS2* levels are essential for the formation of distal metastases in the lungs and liver. In summary, our results indicate that elevated *RRAS2* expression is critical not only as an initial event to trigger tumor formation but also as a continuous requirement to sustain tumor cell properties, including a high proliferation rate, the ability to form tumors in vivo, and the capacity to disseminate and produce metastases.

Another question we sought to address was whether overexpression of wild-type *RRAS2* is accompanied by other gene alterations in breast tumors generated in mice overexpressing *RRAS2*. To investigate this, we analyzed our previous RNA-seq data and searched for mutations present in the mRNA [35]. We identified a total of 5,212 mutations across 544 genes in the 13 independent tumor samples analyzed. A highly conserved pattern of accompanying mutations was observed in various mRNA regions. Notably, certain genes—such as claudin 10 (*CLDN10*), *HSPA9*, and *EGR1*—exhibited missense mutations in their coding regions, which were present in the majority of tumors. Interestingly, other genes not only carried missense mutations in different tumors but also harbored multiple mutations consistently found across samples. These genes included *ARHGAP31*, *HERC6*, *HJURP*, and *IQGAP2*. All those genes have been found mutated in human breast cancers (cBioportal.org) and to be relevant for this disease. For instance, *Egr1* is a tumor suppressor that activates *Tp53* and *Tgfb* expression and has a prognostic value for human BC progression [66]. The *Cldn10* gene encodes a protein found at tight junctions in the plasma membrane and has a SNP associated with an increased risk of BC [67]. The *Hspa9* gene encodes a heat shock protein known as mortalin that plays a fundamental role in mitochondrial biogenesis. Its overexpression correlates with shorter survival in different cancers, including BC [68]. One of the mechanisms could be the sequestering of cytoplasmic proteins, including p53. Although longitudinal studies are necessary to confirm the principles of natural selection, the convergent accumulation of these accompanying mutations across the thirteen independent tumor samples suggests a Darwinian selection process favoring the best-fitted tumors [69].

The RNA-seq data has also been used to infer pathways based on the identity of mutated genes, even though the consequences of most mutations remain unknown, as well as on mRNA expression levels. These analyses have revealed features of RAS signaling, including activation of the MAPK and PI3K/Akt/mTORC1 pathways, as well as Wnt pathway signaling, cancer cell stemness, regulation of the actin cytoskeleton, and interaction with the ECM. Interestingly, a pattern of cancer cell stemness, interactions with the ECM and regulation of the actin cytoskeleton has also emerged from the analysis of the R-RAS2 interactome in a freshly-isolated breast tumor caused by *RRAS2* overexpression. We hypothesized that R-RAS2 might be signaling downstream of BC cell plasma membrane receptors by directly interacting with them.

This hypothesis stems from our previous findings that, in T and B lymphocytes, R-RAS2 directly interacts with T-cell and B-cell antigen receptors (TCR and BCR), promoting homeostatic proliferation and survival signals [48]. Additionally, R-RAS2 constitutively interacts with the B-cell receptor (BCR) in leukemic cells from mice with chronic lymphocytic leukemia (CLL) induced by *RRAS2* overexpression [36]. More recently, we have shown that R-RAS2 exhibits high-affinity binding to immunoreceptor tyrosine-containing activation motifs (ITAMs) in the cytoplasmic tails of BCR and TCR subunits [64]. This interaction promotes GDP-by-GTP exchange in R-RAS2, leading to its activation. Given that tyrosine- and leucine-containing motifs are common in membrane receptors, we hypothesized that R-RAS2 might also associate with plasma membrane proteins in BC cells, thereby participating in signal transduction. Indeed, our proteomic approach has identified several receptors interacting with R-RAS2 and known to impact BC development or malignant behavior, including receptor tyrosine kinase Epha2, β-catenin 1, α-catenin, Ptk7, and CD44. R-RAS2 also interacts with solute carriers (such as GLUT1 and CD98/LAT1) and proteins involved in GPCR signaling. Of these candidates, we prioritized the investigation of R-RAS2 signaling via CD98/LAT1 and CD44.

To explore these interactions, we generated a cell line from a primary TNBC tumor in RRAS2-overexpressing mice and established a knockdown cell line with a fourfold reduction in R-RAS2 protein levels. Additionally, we utilized the *RRAS2*-overexpressing human TNBC cell line BT-549 to corroborate findings. In previous studies using the human MDA-MB-231 BC cell line, we demonstrated that *RRAS2* knockdown decreases phosphorylation within the PI3K/Akt/mTORC1 pathway, indicating R-RAS2’s role in BC cell metabolism and protein translation [47]. Here, using the murine cell line CBM-MBC21, we further confirm these findings. Since CD98/LAT1 is a known activator of mTORC1 through Rag family GTPases, and given that *RRAS2* knockdown reduced mTORC1 activity, we hypothesize that R-RAS2 could function upstream in this pathway by mediating signals from CD98/LAT1. Amino acid depletion experiments, along with phosphorylation analyses downstream of mTORC1 upon amino acid reconstitution, confirm that R-RAS2 is required for CD98/LAT1-mediated mTORC1 activation and BC cell metabolic processes.

The second plasma membrane receptor examined here is CD44, due to its association with cancer stem cell characteristics [20–22]. The standard splicing form, CD44s, essential for epithelial-mesenchymal transition (EMT) and BC progression [70], was found to interact with R-RAS2 in murine CBM-MBC21 TNBC cells. We assessed the effect of R-RAS2 depletion on migration in both human and murine BC cell lines, promoted through CD44 interaction with hyaluronic acid, an extracellular matrix component. CD44’s binding to hyaluronic acid induces conformational changes, enabling intracellular binding of adaptor proteins and cytoskeletal components that activate multiple signaling pathways related to cell proliferation, adhesion, migration, and invasion [23]. During EMT, cancer cells, often expressing elevated CD44, acquire stem cell-like properties and exhibit increased invasiveness [24]. Our results show that R-RAS2 co-localizes with CD44 at cell projections with active actin polymerization and Akt phosphorylation. Furthermore, using a FRET biosensor to detect GTP-bound R-RAS2, we demonstrate its activation at protruding ends, dependent on CD44-hyaluronic acid interaction. These findings suggest that R-RAS2 associates with CD44 at the BC cell plasma membrane to promote actin cytoskeleton rearrangement, cell migration, and metastasis formation. We also show that sustained RRAS2 expression in murine CBM-MBC21 cells is necessary for metastasis from the primary breast tumor to the lungs and liver.

Beyond supporting BC cell migration and metastasis, CD44 is a known marker and promoter of cancer cells stemness [21, 22, 71]. Interestingly, another membrane protein found here in the R-RAS2 interactome is Epcam, which is also an important breast cancer stem cell marker [72]. Upstream of BC stem cell markers is the Wnt pathway, both the canonical β-catenin-dependent and the alternative, especially in TNBC [25]. In this manuscript we have identified β-catenin, α-catenin and Ptk7 as interactors of R-RAS2 that are involved in the Wnt signaling pathway. In addition, our GOBP analysis of somatic mutations in *RRAS2* overexpression-induced murine TNBC has identified an effect on the Wnt pathway, whereas our IPA analysis of mRNA expression has identified an effect on both the canonical Wnt and the alternative Wnt/Ca2 + pathways. Furthermore, frizzled class receptor 6 (*FZD6*) is a Wnt receptor that has been found bearing missense mutations in 6 out of the 13 *RRAS2* induced mouse tumors that have been sequenced here. All those data are consequent with R-RAS2 having a role in Wnt pathway signaling in TNBC and having and effect on BC stemness and chemoresistance. While the impact of R-RAS2 on Wnt pathway activation remains to be examined, this link warrants further research.

In addition to the functional relationship between RRAS2 and the Wnt pathways, there are many other findings that are worh to be studied in following studies. One is the high number of tubulins that has been detected in the R-RAS2 interactome and that produces a KEGG fingerprint of interaction with motor proteins. Another, is the striking number of non-classical Qa-2 class I MHC chains detected in the R-RAS2 interactome. Since expression of this MHC-I has been found to be excluded from breast cancer stem cells [73], one may suspect that R-RAS2 could be involved in the downregulation of Qa-2.

The observed associations between R-RAS2, CD44, and Wnt receptors align with the higher R-RAS2 expression levels reported in postpartum TNBC cases, which are often associated with worse prognoses compared to other BC types and diagnoses in older or nulliparous women [35]. Currently, therapeutic strategies targeting upstream activators and downstream pathways of R-RAS2, such as PI3K/Akt/mTOR, are being explored for TNBC treatment [74]. Additionally, direct R-RAS2 inhibitors could provide a targeted treatment approach for cancers overexpressing *RRAS2*, including TNBC, CLL, and potentially other malignancies.

## Conclusions

In conclusion, our study demonstrates that R-RAS2, a GTPase of the RAS-related subfamily, is physically and functionally associated with various plasma membrane receptors in BC cells, influencing critical cancer cell traits such as proliferation, metabolism, migration, extracellular matrix interactions, and distal metastasis formation. We show that R-RAS2 mediates mTOR activation via the CD98/LAT1 transporter of large neutral amino acids and promotes actin cytoskeleton rearrangement, cell projection formation, migration, invasion, and metastasis through the hyaluronic acid receptor CD44.

## Electronic supplementary material

Below is the link to the electronic supplementary material.

Supplementary Material 1

Supplementary Material 2

Supplementary Material 3

Supplementary Material 4

Supplementary Material 5

Supplementary Material 6

Supplementary Material 7

Supplementary Material 8

Supplementary Material 9

## Data Availability

No datasets were generated or analysed during the current study.
