# Supplementary material for: The R-RAS2 GTPase is a signaling hub in triple-negative breast cancer cell metabolism and metastatic behavior
Source: J Hematol Oncol. 2025 Apr 12;18:41. doi: 10.1186/s13045-025-01693-3 (PMC11993990; doi:10.1186/s13045-025-01693-3)
Supplement: Supplementary file 9 — Supplementary Material 9 [file 13045_2025_1693_MOESM9_ESM.docx]

**EXTENDED DATA FIGURE LEGENDS**

**
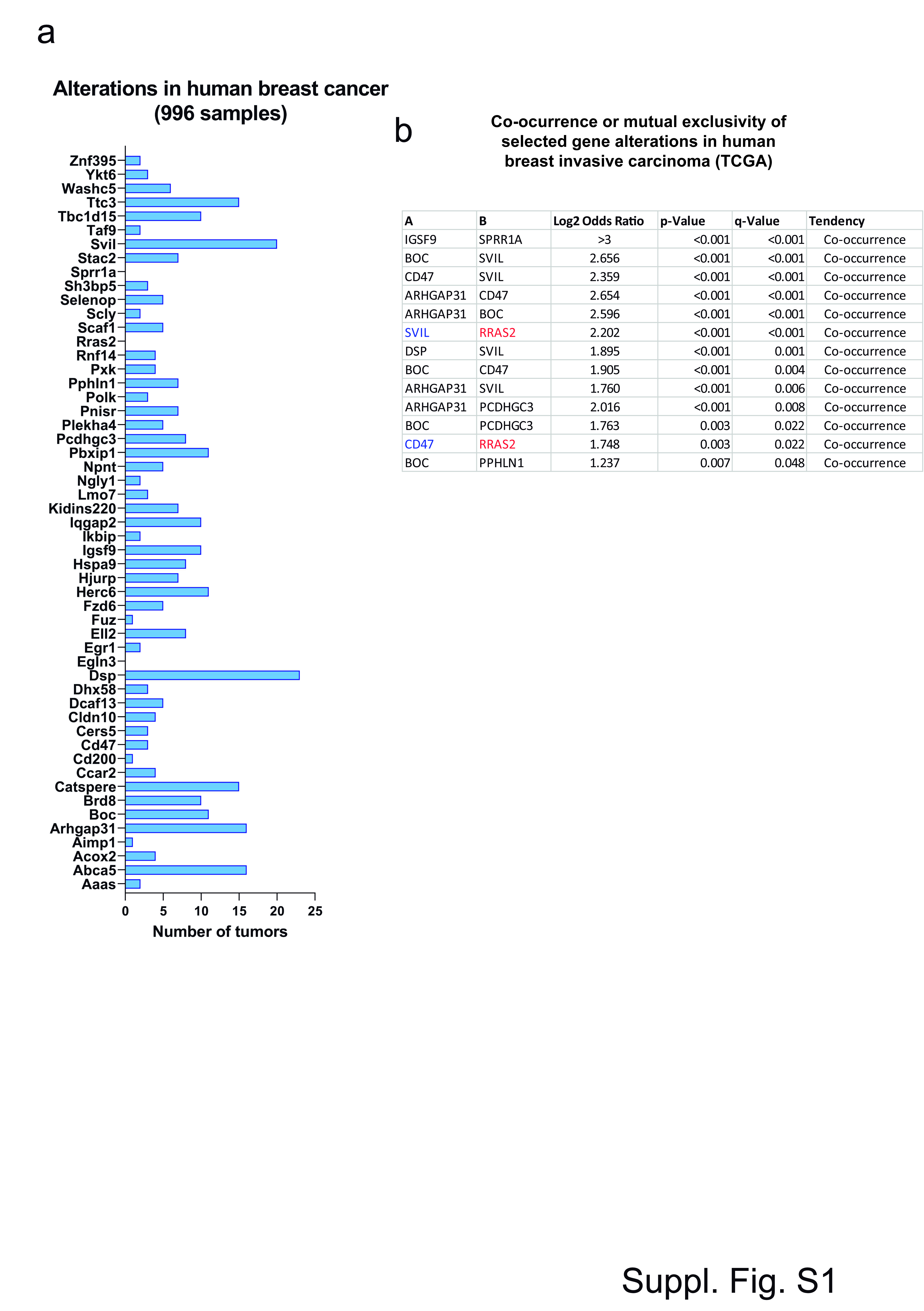
**

**Extended Data Fig. S1. a,** Bar plot showing the number of breast cancer samples in The Cancer Genome Atlas database (n=996 samples) found to bear mutations in the indicated genes of Fig. 1f. The analysis was carried out using the cBioportal.org platform. **b,** Co-ocurrence or mutual exclusivity for gene mutations taken two-by-two from the list of Fig. S1a of 996 BC samples in TCGA and analyzed using cBioportal.org.

**
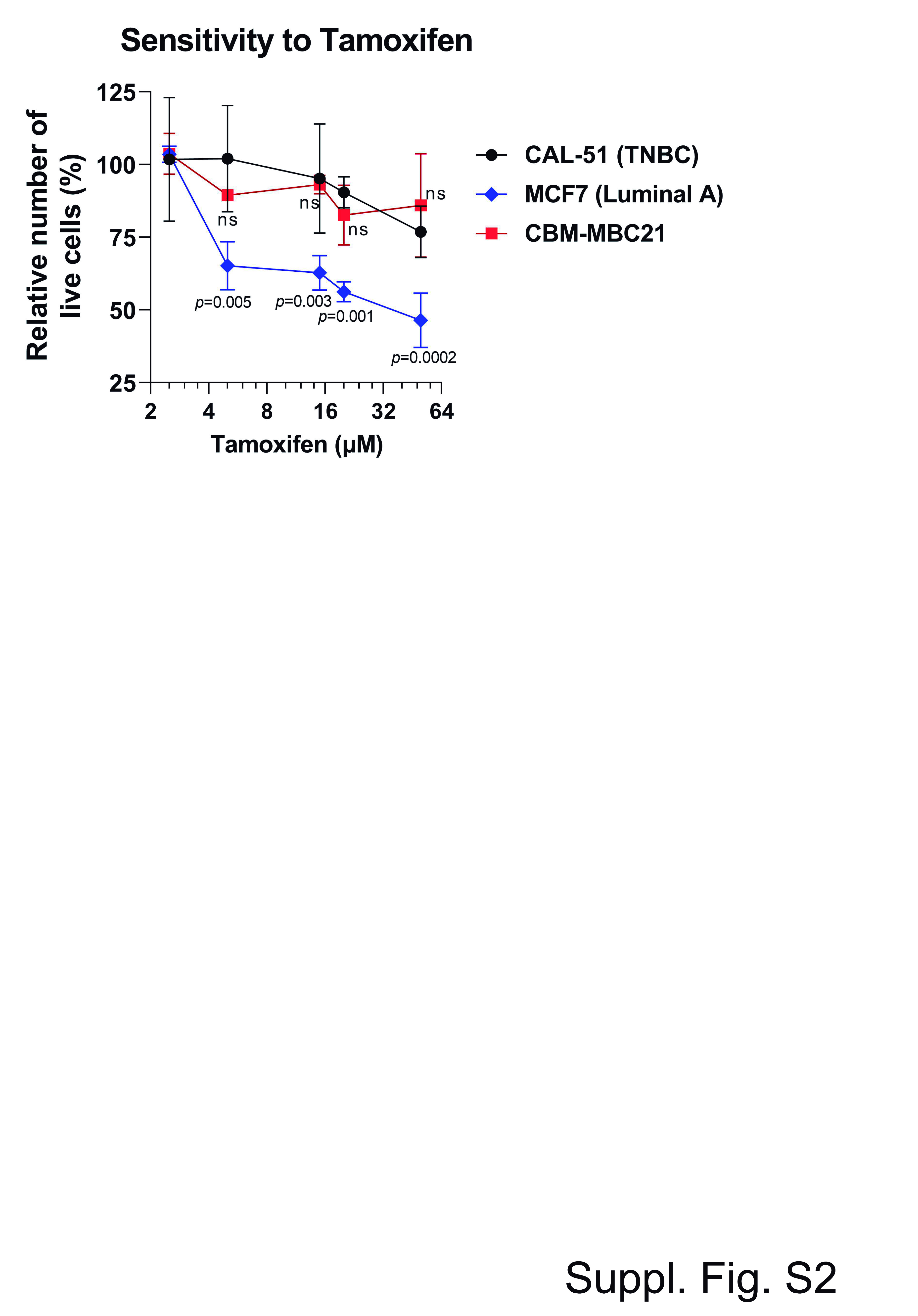
**

**Extended Data Fig. S2.** Sensitivity to different concentrations of tamoxifen was tested for the CBM-MBC21 cell line in parallel to the human TNBC cell line CAL-51 and the human luminal A cell line MCF7. The effect of tamoxifen is shown for each cell line relative to the number of live cells in the absence of tamoxifen, taken as 100%. Datapoints are represented as the mean±s.e.m. of triplicates. Significance was assessed for each cell line using a one-way ANOVA test. ns, not significant, *p* > 0.05.


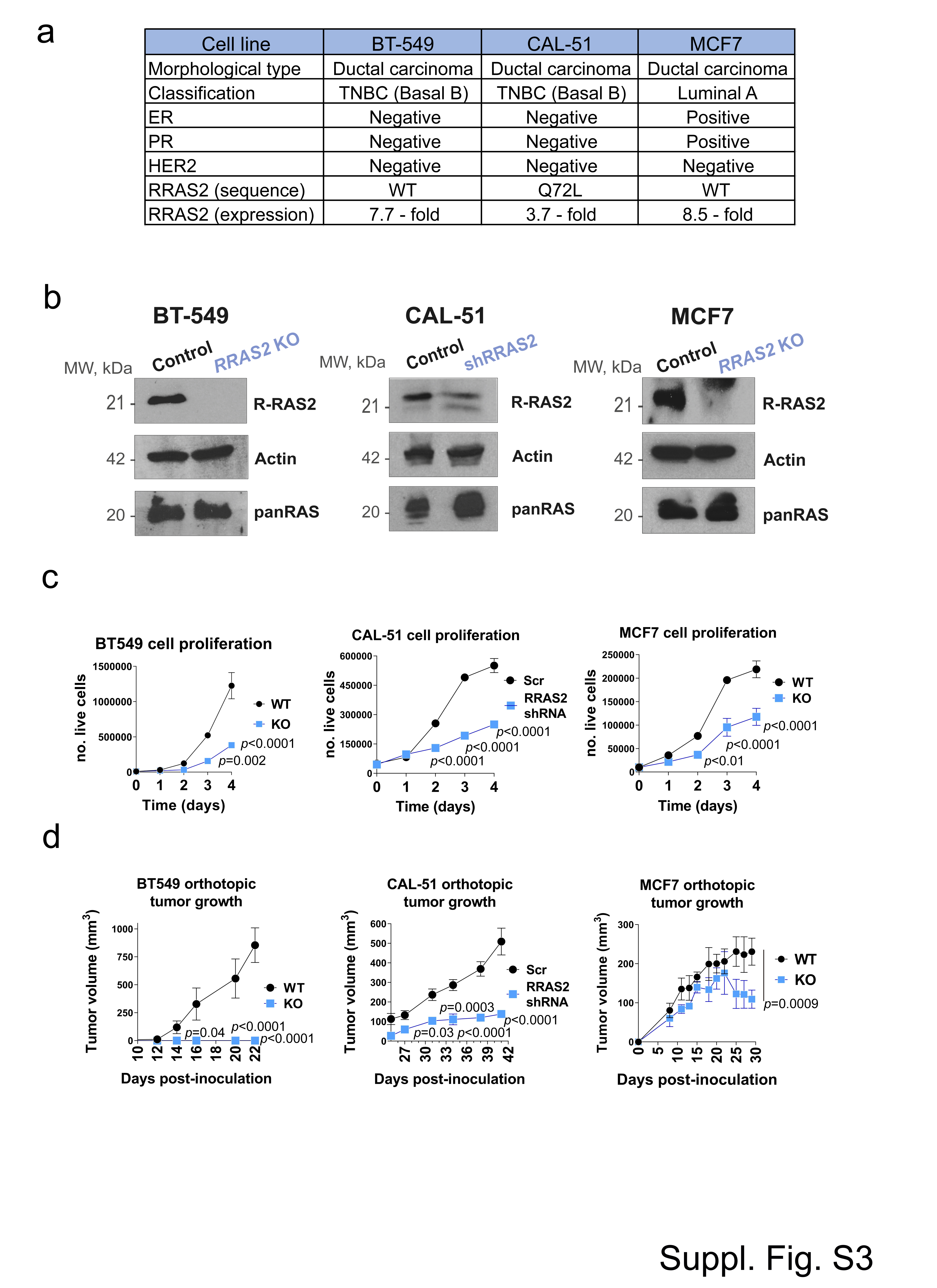


**Extended Data Fig. S3. R-RAS2 is necessary for both *in vitro* proliferation and growth at an orthotopic location of human breast cancer tumors.** **a,** Characteristics of the three human breast cancer cell lines used for the *in vitro* and *in vivo* studies. *RRAS2* expression was measured by RT-qPCR of mRNA and normalized to the average of mRNA expression in 10 samples of human breast non-tumoral epithelial tissue. **b,** Western blot analysis of R-RAS2 protein expression in BT-549, CAL-51 and MCF7 parental cells and RRAS2-deficient cells generated either by CRIPR/Cas9 (KO) or shRNA technologies, as indicated. Loading and specificity controls were carried out by incubation with an anti-actin and an anti-pan-RAS (classical) antibody, respectively. **c,** *In vitro* proliferation of wild type BT549, CAL-51 and MCF7 breast cancer cells in comparison with their corresponding *RRAS2* knockout (BT549, MCF7) or knockdown (CAL-51) cell lines. Datapoints represent the mean±s.e.m. of triplicates. Significance was assessed by two-way ANOVA tests. **d,** *In vivo* tumor growth in orthotopic location of CAL-51 control or CAL-51 *RRAS2* knockdown cells (4 × 10^6^), or MCF7 control, MCF7-*RRAS2* KO, BT-549 control or BT-549-*RRAS2* KO cells (5 × 10^6^) injected into the left inguinal mammary gland of *Rag2*^−/−^γc^−/−^ female mice. Datapoints are represented as the mean±s.e.m. for *n*=5 (BT549 and MCF7) and n=7 (CAL-51) mice per group. Significance was assessed by two-way ANOVA tests.

**
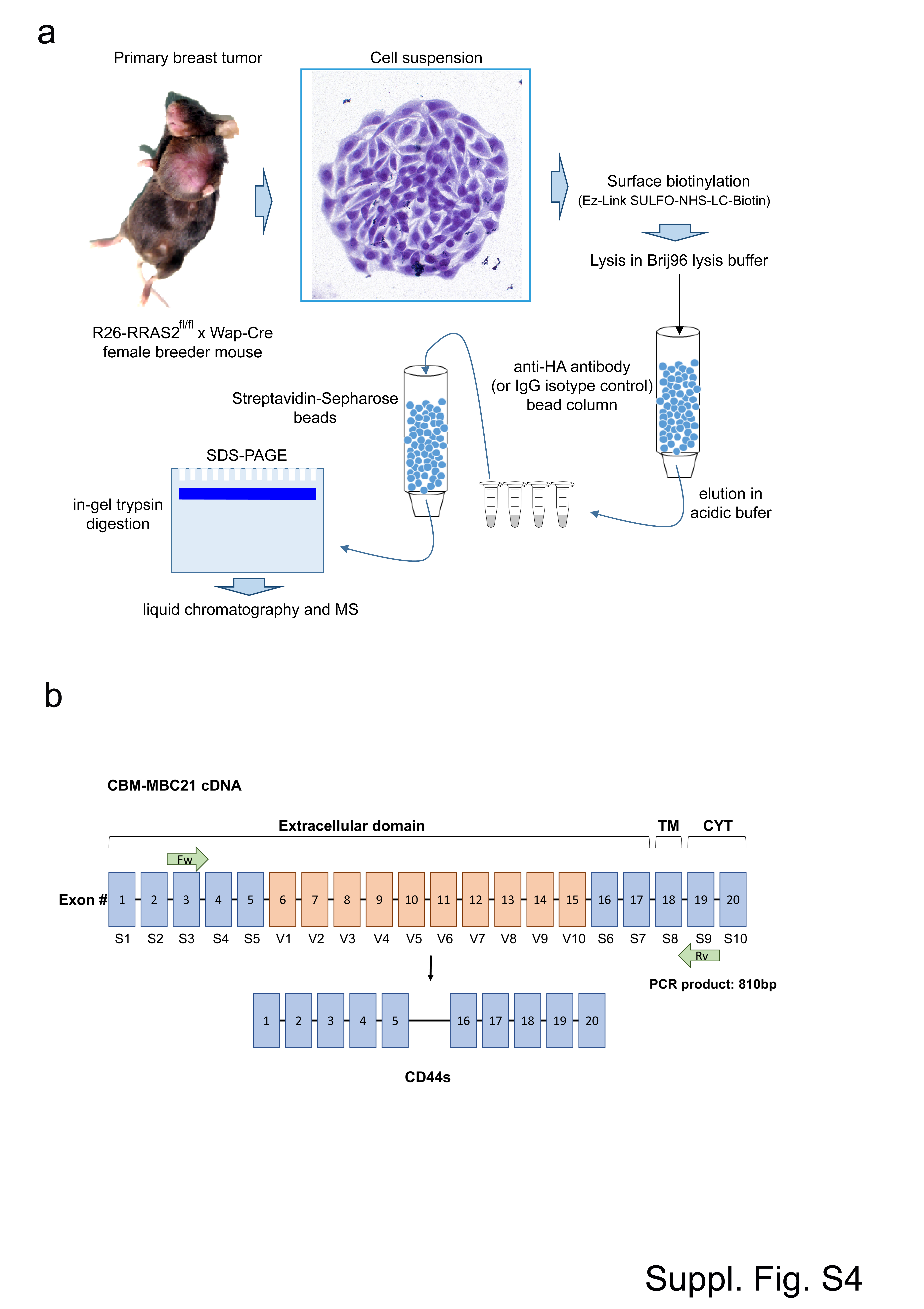
**

**Extended Data Fig. S4. a,** Cartoon of the purification and identification of plasma membrane proteins that interact with R-RAS2 in a primary mouse breast tumor found in a R26-*RRAS2*^fl/fl^ x Wap-Cre female mouse breeder. **b,** Scheme of the exon organization of murine *Cd44* and their corresponding translation into protein domains. The sites of the forward (Fw) and reverse (Rv) primers annealing to exons 3 and 9, respectively, used for sequencing are indicated. The exons present in the CD44s isoform found in CBM-MBC21 cells are indicated in blue and those spliced out are in beige; TM, transmembrane domain; CYT, cytoplasmic tail.

**
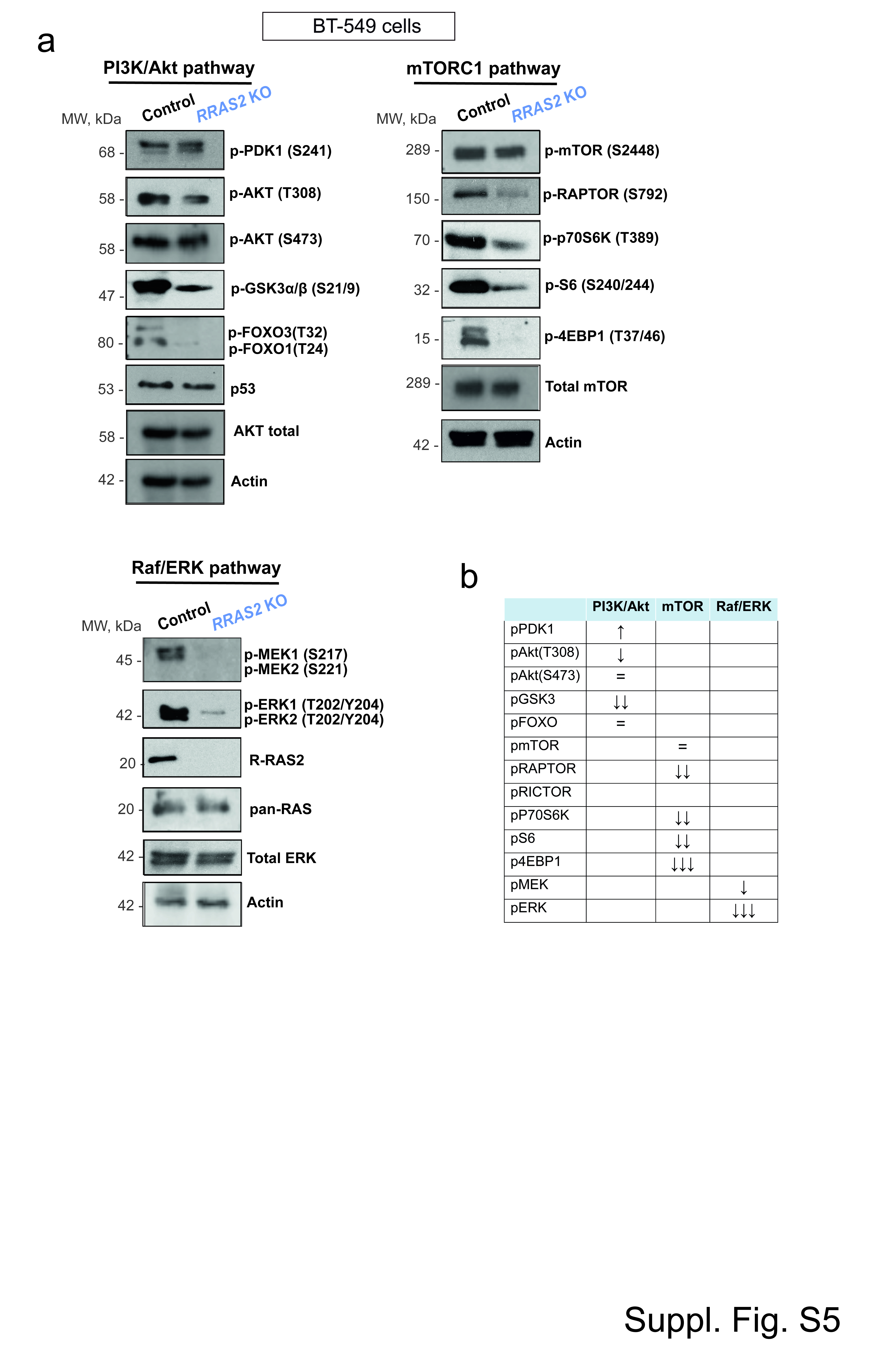
**

**Extended Data Fig. S5. a,** Western blot analysis of signaling pathway activity based on the phosphorylation of key residues in the elements indicated. Post-nuclear cell lysates of wild type BT-549 cells and a CRISPR/Cas9-generated *RRAS2* knockout of that cell line were analyzed in the blots. **b,** Summary of the results generated by Western blot. The inhibitory effect of R-RAS2 depletion is indicated by the number of arrows pointing downwards.

**
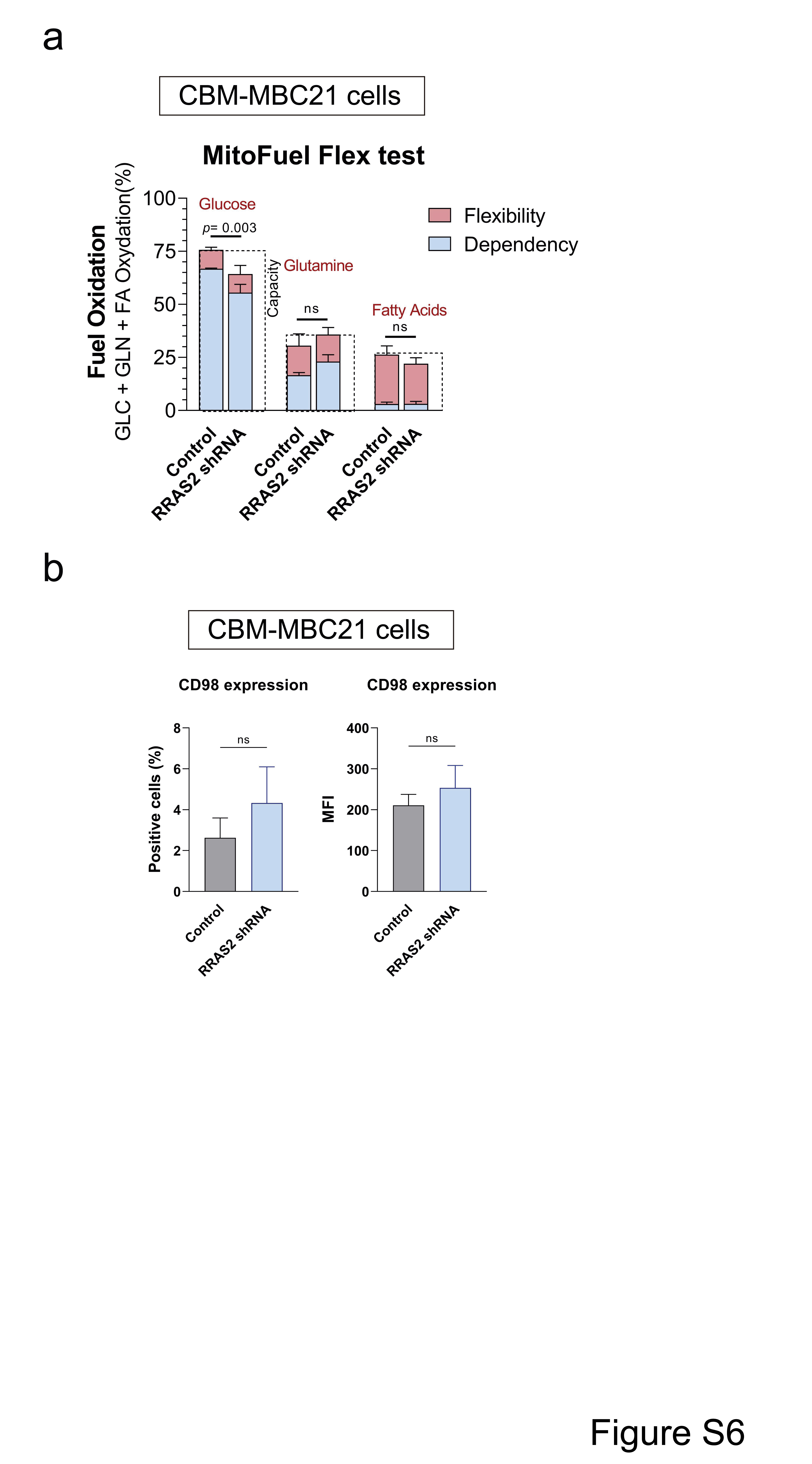
**

**Extended Data Fig. S6. a,** MitoFuel Flex Test on CBM-MBC21 based on the measurement of oxygen consumption rate (OCR) in basal conditions after inhibiting the use of the indicated substrates (glucose, glutamine or fatty acids) by using specific inhibitors. Dependency refers to the impact on OCR of inhibiting the use of the indicated substrate; Capacity refers to the capacity of the mitochondria to sustain OCR by using the indicated substrate when the use of the other two is blocked; Flexibility is the result of substracting Dependency to Capacity and is the increase in OCR by oxidizing the indicated substrate when the use of the other two substrates is blocked. **b,** Bar plots showing the mean±s.e.m. of CD98 expression by CBM-MBC21 BC cells both as the mean fluorescence intensity (MFI) and as the percentage of CD98+ cells calculated by flow cytometry. Statistical significance was assessed by carrying out Mann-Whitney tests. ns, not significant, p>0.05.


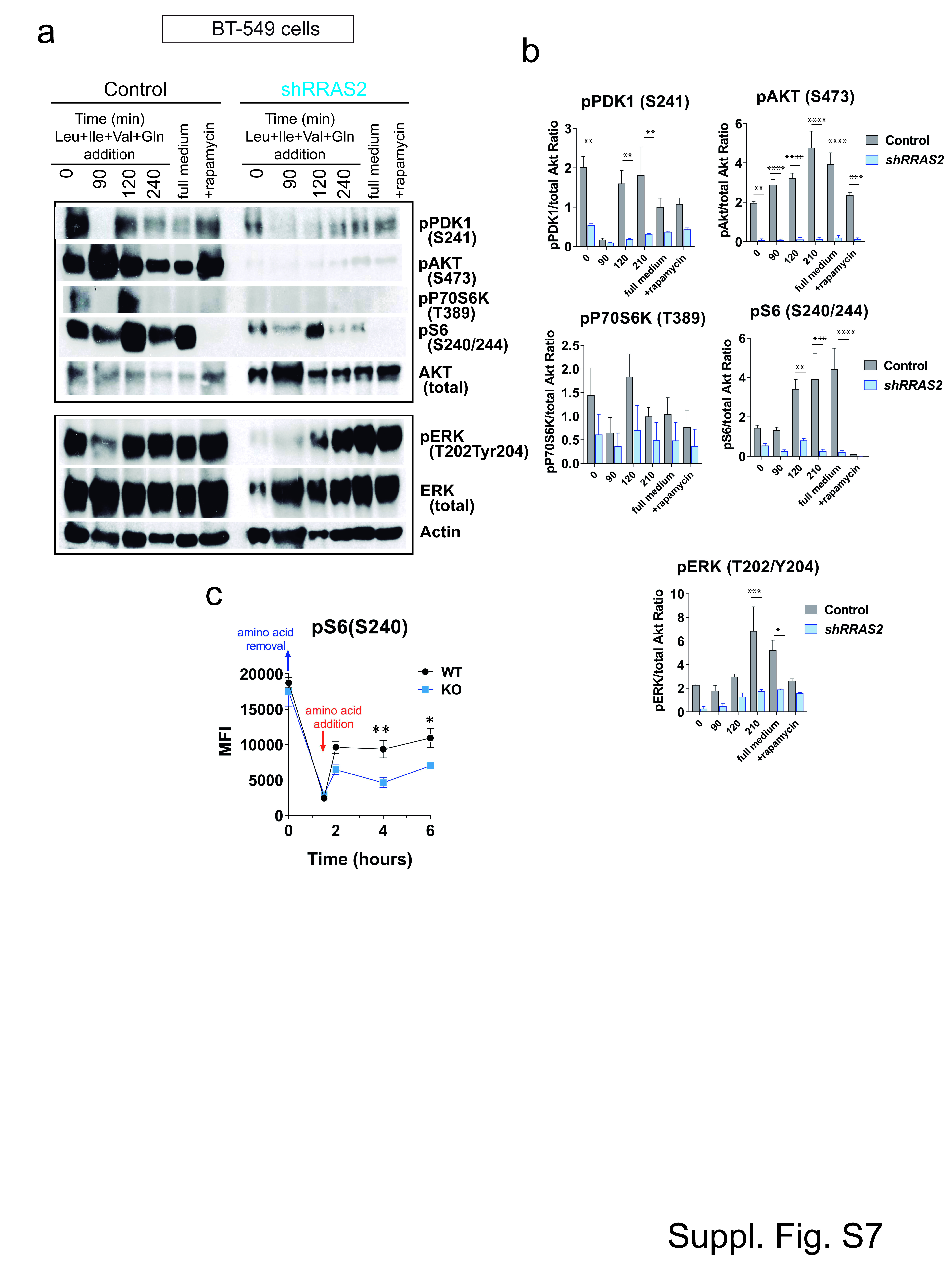


**Extended Data Fig. S7. a,** The effect of R-RAS2 depletion on the capacity of BT549 BC cells to activate the mTORC1 pathway in response to hydrophobic long-chain amino acids was measured by Western blot analysis. Control and RRAS2 knockout BT-549 cells were deprived of the amino acids Leu, Ile, Val, and Gln for 1.5 hours, followed by replenishment of the specified amino acids for the indicated time points (in minutes). Post-nuclear cell lysates were analyzed via Western blotting. As controls, lysates from cells maintained in full medium throughout the experiment and from cells treated with 50 nM rapamycin in full medium were included. **b,** Quantification by densitometry of Western blots as in Fig. S7a run in triplicate. Bar plots show the mean±s.e.m of densitometry data referring the intensity of the phosphoprotein bands to that of total Akt. Significance was assessed using a two-way ANOVA test. *, *p*<0.05; **, *p*<0.01; ***, *p*<0.001; ****, *p*<0.0001. **c,** Effect of amino acids Leu, Ile, Val and Gln deprivation followed by replenishment on the phosphorylation of S6 protein at Ser240 analyzed by flow cytometry. Datapoints represent the mean±s.e.m of triplicates. Significance was assessed using a two-way ANOVA Sidak’s multiple comparison test. *, *p*=0.02; **, *p*=0.005.


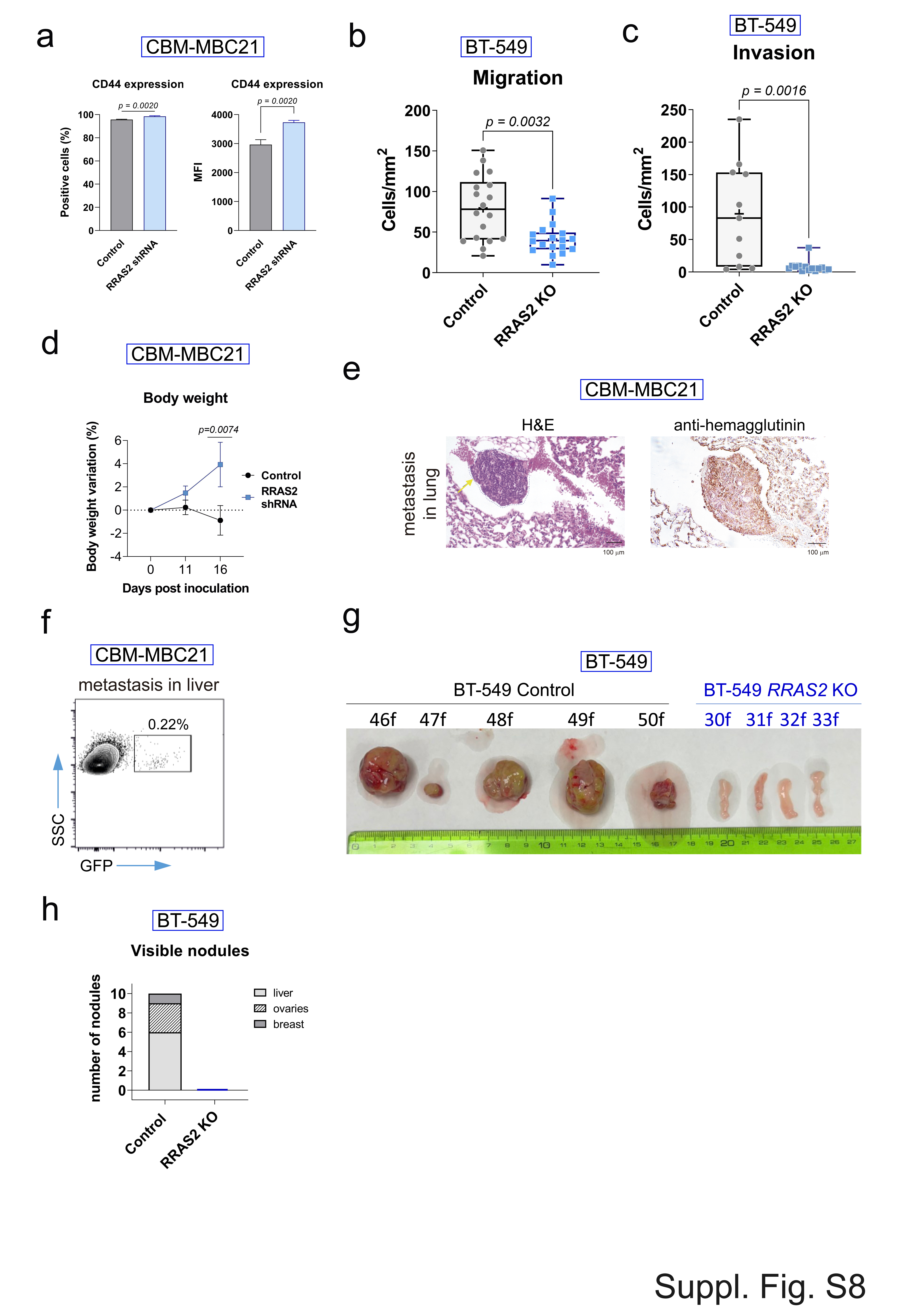


**Extended Data Fig. S8. a,** Bar plots showing the mean±s.e.m. of CD44 expression by CBM-MBC21 BC cells both as the mean fluorescence intensity (MFI) and as the percentage of CD44+ cells calculated by flow cytometry. Statistical significance was assessed by carrying out Mann-Whitney tests. **b,** Box and whiskers plot showing a migration assay carried out with BT-549 human BC cells as in Fig. 8a. **c,** Box and whiskers plot showing an invasion assay carried out with BT-549 human BC cells as in Fig. 8b. **d,** Relative body weight variation from day 0 of CBM-MBC21 cell inoculation in the left inguinal mammary gland to day 16 post-inoculation. Data is expressed as the mean±s.e.m. Significance was assessed using a two-way ANOVA test. **e,** Hematoxilin and eosin (H&E) staining of lung sections from a mouse inoculated with control and CBM-MBC21 cells, as in Fig. S7d, chosen to illustrate the presence of a metastatic nodule (yellow arrow) in the lung parenchyma. An immunoperoxidase staining with an anti-Hag of the same nodule is shown to underscore the overexpression of R-RAS2. **f,** Two-parameter plot illustrating the identification of metastatic CBM-MBC21 cells in the liver according to the side-scatter and the expression of GFP. **g,** Photographs of the primary tumors found at day 39 post-inoculation of female RAG2^-/-^γc^-/-^ mice in the left inguinal mammary gland with control and knockout BT-549 cells. **h,** Number of tumor nodules detected upon visual inspection of the liver, ovaries and breast of female RAG2^-/-^γc^-/-^ mice inoculated as in Fig. S8g.
